# Social prescribing outcomes: a mapping review of the evidence from 13 countries to identify key common outcomes

**DOI:** 10.3389/fmed.2023.1266429

**Published:** 2023-11-07

**Authors:** Jill Sonke, Nico Manhas, Cassandra Belden, Jane Morgan-Daniel, Seher Akram, Stefany Marjani, Oluwasanmi Oduntan, Gabrielle Hammond, Gabriella Martinez, Gray Davidson Carroll, Alexandra K. Rodriguez, Shanaé Burch, Aaron J. Colverson, Virginia Pesata, Daisy Fancourt

**Affiliations:** ^1^Center for Arts in Medicine, College of the Arts, University of Florida, Gainesville, FL, United States; ^2^Health Science Center Libraries, University of Florida, Gainesville, FL, United States; ^3^College of Medicine, University of Lagos, Lagos, Nigeria; ^4^College of Public Health and Health Professions, University of Florida, Gainesville, FL, United States; ^5^School of Music, College of the Arts, University of Florida, Gainesville, FL, United States; ^6^Department of Behavioural Science and Health, University College London, London, United Kingdom

**Keywords:** social prescribing, arts prescribing, outcomes, social prescribing outcomes, mapping review

## Abstract

**Introduction:**

As a means for supporting a range of health and wellbeing goals, social prescribing programs have been implemented around the world. Reflecting a range of contexts, needs, innovation, and programing, a broad array of outcomes has been studied in relation to these programs. As interest in social prescribing grows, more targeted study of key outcomes and in turn evidence synthesis that can inform evidence-based practice, policy, and investment is needed.

**Methods and Results:**

This mapping review identified, described, and synthesized the broad array of social prescribing outcomes that have been studied in 13 countries and maps the outcomes that have been most commonly studied. From 87 articles included in this review, a total of 347 unique outcomes were identified, including 278 unique patient outcomes and 69 unique system outcomes. The most commonly studied categories of patient outcomes were found to be mental health, lifestyle and behavior, and patient/service user experience. The most commonly studied system outcomes were healthcare/service utilization and financial/economic outcomes.

**Discussion:**

This review highlights the value of heterogeneity and mixed methods approaches in outcomes studies for capturing nuanced experiences and outcomes in this nascent area of practice, while contributing to the advancement of evidence synthesis for social prescribing globally by quantifying and offering insight into the outcomes that have been studied to date. It also lays a foundation for the development of key common outcomes and a Core Outcomes Set for social prescribing. Additionally, it identified key outcomes that, given their relationship to critical health and social issues, warrant both broader and deeper study.

## 1. Introduction

Social prescribing (SP) programs are increasingly being implemented in nations throughout the world. Social prescribing has been defined as “a means for trusted individuals in clinical and community settings to identify that a person has non-medical, health-related social needs and to subsequently connect them to non-clinical supports and services within the community by co-producing a social prescription–a non-medical prescription, to improve health and well-being and to strengthen community connections” (1). SP programs seek to address social determinants of health or underlying and systemic causes of health issues and inequities, to fill the gap between clinical and non-clinical services, and to broaden the landscape of health promotion to include local community-based resources. It recognizes that individuals have social needs related to health that can be addressed in their community.

SP programs have been implemented–at various levels of scale - in at least 17 nations, including the United Kingdom, Australia, Canada, Ireland, Japan, New Zealand, Portugal, Singapore, and the United States (2, 3). Studies of these programs examine a wide range of outcomes. While this heterogeneity reflects the wide range of innovation and practices involved in this relatively nascent arena of practice and policy, it limits evidence synthesis and leaves the depth of social prescribing’s impact yet to be identified on a wide scale. As these programs proliferate at an increasing pace throughout the world today, the need for evidence synthesis to inform evidence-based practice, policy, and investment is critical. Further, as SP programs are implemented in a wider array of nations and socio-political, cultural, healthcare and economic contexts, there is increasing need for culture- or country-specific evidence synthesis that can advance culturally appropriate practice and policy in those areas.

In efforts to advance and strengthen the evidence base, several studies to date have investigated and documented outcomes studied in relation to social prescribing programs in specific regions, notably in the United Kingdom (UK). Polley et al. (4) reported on 14 papers published between 2000 and 2017, collating outcomes relating to demand for general practitioner (GP) services, accident and emergency attendance, demand for other secondary care services, value for money assessment such as cost–benefit and return on investment, and social return on investment. Polley et al. (5) subsequently built on this work by reviewing social prescribing outcomes literature published between February 2017 and March 2018. A resulting publication presented 67 unique outcomes found in the literature, up to 2018, and organized them into 6 categories–general (included wellbeing, quality of life, and social connectedness), physical, psychological, welfare, spiritual, and social (4).

This mapping review aimed to advance and expand this work undertaken in the UK by identifying, describing, and synthesizing the broad array of social prescribing outcomes that have been studied in the 13 countries cited in the World Health Organization’s Social Prescribing Toolkit (2). Additionally, it sought to identify the outcomes that have most commonly been studied as a step toward developing a set of key common outcomes for social prescribing in the United States (US) and establishing an outcomes framework for advancing related research. This work recognizes that, given very different social/political structures and health systems of the US and UK, where the majority of social prescribing research has been done, specific priority outcomes should be explored and identified for the US. This work also seeks to lay groundwork for future development of a formal core outcomes set (COS) for social prescribing.

## 2. Materials and methods

A mapping review was selected because this methodology takes a broad approach to categorizing and contextualizing elements of existing literature on a topic (6). Mapping reviews are used to create systematic maps of evidence domains, through which quantitative analysis of evidence gaps can occur and recommendations for future research or reviews can be made (7). Mapping reviews are a subset of scoping reviews, in that they use both systematic and iterative processes to search the literature. Although the same reporting guidelines are used for both (8), mapping reviews tend to describe the research field overall versus the detailed content of specific studies, so that theoretical connections can be made and practice-relevant questions for future research or reviews can be posed more easily (9).

This review’s purpose was to map the commonly studied outcomes for social prescribing in 13 countries. While the focus of this review was on quantifying the most commonly studied outcomes in relation to social prescribing in these countries, it also extracted key data points, such as geographic locations. This review did not seek to report on demographic characteristics of the populations studied, research and evaluation methods or measures, or the efficacy of social prescribing programs. However, a few methods and other details are noted in the description of studies noted as examples in the results sections below.

### 2.1. Definitions

In keeping with Polley et al. (5), this mapping review defines an outcome as “something that is expected to change from the result of an intervention” (5, 10); it defines social prescribing as the referring of individuals by care providers to non-clinical activities in their community to support their health and wellbeing.

### 2.2. Search strategy

The review builds on a previous study of social prescribing outcomes in the United Kingdom (11). With the permission of its authors, the search strategy from the United Kingdom study was adapted by a health sciences librarian for this review’s research question, “What are the key outcomes reported for social prescribing interventions in Australia, Canada, Ireland, Japan, New Zealand, Portugal, Singapore, the United Kingdom, China, and the United States?” A Population, Concept, Context (PCC) conceptual framework (see below) was used to develop the search strategy and eligibility criteria for this review.

Preliminary test searching to inform the development of the search strategy took place in December 2022, using the databases PubMed and Web of Science. Following feedback from the research team on the search results, the final search strategy was created and translated into eight databases using available subject headings, truncated and phrase-searched keywords in the title and abstract fields, and language limits. The final literature search occurred on January 20, 2023, in the following databases: CINAHL (EBSCO), PsycINFO (EBSCO), Psychology and Behavioral Sciences Collection (EBSCO), Sociological Collection (EBSCO), Embase (Elsevier), Scopus (Elsevier), Web of Science (Clarivate Analytics), and PubMed. A sample search strategy for PubMed is available as a Supplementary material. This same strategy was adapted to the different search formats of the other databases.

Handsearching of numerous resources also occurred between February 13–29, 2023, to gather any gray literature not included in the bibliographic databases. Hand searches included snowballing of the systematic and other reviews captured in the database searches, searches of web archives and databases maintained by the University of Florida Center for Arts in Medicine (including the Arts in Health Research Database), the Social Prescribing Network, and the National Academy for Social Prescribing.

### 2.3. Inclusion and exclusion criteria

Inclusion criteria were based on a Population, Concept, Context (PCC) conceptual framework, and also included additional criteria, as noted in Table 1.

**Table 1:** Inclusion criteria.

Population/Location	Studies involving human populations (including providers and patients) in Australia, Canada, Ireland, Japan, New Zealand, Portugal, Singapore, United Kingdom (England, Scotland, Wales and Northern Ireland), China (western pacific region), or the United States.
Concept	Social prescribing as an intervention, defined as *the referring of individuals by care providers to non-clinical activities in their community to support their health and wellbeing,* and including a referral mechanism from a healthcare system or provider (i.e., a link worker, community health worker, patient navigator, care navigator or similar role or mechanism for facilitating referrals and/or prescriptions).
Context	Outcomes reported by or on behalf of participants and systems involved in social prescribing interventions.
Type of evidence	All literature types, including original research studies, evidence synthesis reviews, reports, and gray literature.
Source of evidence	Peer-review journal or other credible sources including universities, professional organizations, governmental, and global organizations (i.e., the World Health Organization).
Date range	Any year through 2023.
Outcome reported	Reported outcomes (positive, neutral, or negative) related to the impact of social prescribing.
Use of measures	Evidence of defined measures used to arrive at outcomes (qualitative, quantitative, mixed-methods, etc.).
Language	English, Chinese, Japanese, and Spanish

Reviews were included in the search to provide the opportunity for discovery of other syntheses of outcomes as well as studies that–due to lack of common taxonomy and reporting guidelines on the topic of social prescribing–may not have been captured by the database search strategy. However, reviews were not included in data extraction or in the analysis. Studies were excluded if they presented practice models or discussed theory with no outcomes measured. These same inclusion and exclusion criteria were used for title and abstract screening and for full-text screening.

### 2.4. Screening and data extraction

The search results were imported into the screening software Covidence, where automatic de-duplication of the results occurred. Nine members of the research team screened all article titles and abstracts, followed by full-text screening of the remaining articles. Conflicts were resolved by six members of the team. Data were extracted from articles that were included in the review based on the full-text screening. The following data were extracted from each article, where possible:

- Author(s)
- Institutions involved in the work presented
- Disciplines of authors and other partners involved
- Title
- Year of publication
- Journal name
- Journal discipline
- Funding model
- Type of article (i.e., original research, literature review, report, etc.)
- Location(s)
- Study population
- Sample size
- Scope of “social prescribing” used (i.e., social prescribing or arts on prescription)
- Cross-sector partnerships engaged
- Outcomes measured or reported
- Relevant policy cited
- Key challenges noted
- Noted instances of harm or negative events

Following data extraction, outcomes were verified three times (compared against the source article) by six members of the research team, and until no errors or discrepancies were found. Care was taken to list each unique outcome as stated in the articles (reduced to key terms when necessary), even when similar to others. After quantifying both the recurring and non-recurring outcomes, like outcomes were grouped into outcome categories.

Categories were developed in two stages. First, all unique outcomes were placed in a table that categorized same but differently worded outcomes (e.g., accident and emergency visits / emergency visits, GP visits/GP attendance) together in specified columns. Anything that was unlike another outcome was compiled into a “Z” column. In this primary phase of categorization, outcomes were organized under patient outcomes and system outcomes. All members of the analysis team participated in the process. A second, and more complex, phase of categorization developed sub-categories. In this phase, and using an inductive qualitative content analysis approach (12, 13), three members of the research team worked independently and then collaboratively in an iterative process of organizing outcomes into distinct categories based on dialogue and articulation of differences in outcomes across categorical groups.

This article reports primarily on these documented outcomes, along with the geographic locations of the investigations. Subsequent articles will report on other data extraction elements.

## 3. Results

**Figure 1:**
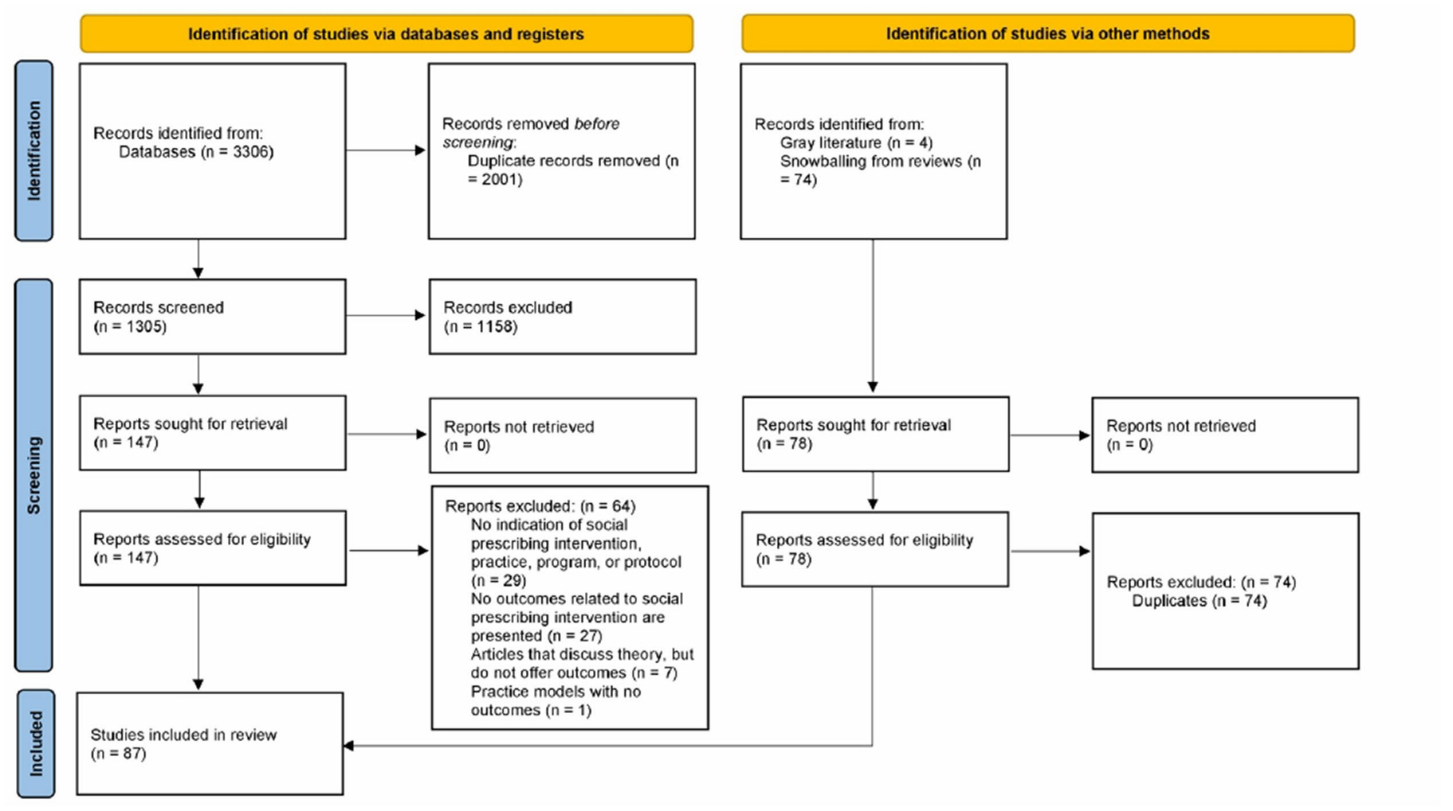
PRISMA 2020 flow diagram foe new systematic reviews which included searches of database, registers and other sources (122).

The bibliographic databases search produced 3,306 results. An additional 78 articles were identified using other methods (gray literature and snowballing). After 2,001 duplicates were removed from the database search results, 1,305 unique references remained. A total of 1,158 references were excluded, leaving 225 eligible studies. A total of 138 studies were excluded with reasons leaving 87 total references for full text review and data extraction (See Figure 1).

Of the 87 included articles, 60 were original research articles, 6 were research protocols, and 21 were reviews (e.g., systematic or scoping reviews). Of the systematic reviews, 4 studies were conducted in the United Kingdom, and single studies were conducted in Canada, Germany, Ireland, Portugal, and the United States. Additional reviews featured studies from Australia, Denmark, the Netherlands, Norway, Scandinavia, South Korea, Sweden, and Taiwan. Some projects began over 30 years ago, with average durations ranging from 3–24 months. Across the systematic reviews, the range of included articles was between 7 (14) and 53 (15). Collectively, the reviews underscore the need for further research, particularly to better understand individual and public health outcomes and cost-benefits. See Table 2 for review type, count, and citation.

**Table 2:** Types of reviews with accompanying citation.

Review type	*N*	Citation
Systematic	9	(14–22)
Literature	3	(23–25)
Narrative	2	(26, 27)
Scoping	2	(28, 29)
Mapping	1	(5)
Qualitative meta-synthesis	1	(30)
Realist	1	(31)
Systematic scoping	1	(32)
Systematized	1	(33)

Snowballing confirmed that all of the articles included in these review articles had also been found in the database searches. To avoid redundancy in quantifying outcomes, data extracted from the review articles were not included in outcome counts.

The majority (*n* = 60) of the articles included in this review featured research or evaluation of social prescribing programs in England. Studies of programs across the UK were presented in 28 articles. While programs in Japan, New Zealand, Singapore, and China were included in the WHO Social Prescribing Toolkit and therefore in this search, no publications reporting outcomes from those countries met criteria for inclusion in the review. See Table 3 for a country count breakdown. Also see Supplemental material for a table that shows the number of papers that presented outcomes, by category, from each country.

**Table 3:** Country breakdown.

Country or region	# of articles reporting outcomes from country/region	Country or region	# of articles reporting outcomes from country/region
Australia	7	Scandinavia	1
Canada	6	Scotland	2
Denmark	2	South Korea	1
England	60	Sweden	1
Ireland	3	Taiwan	1
Netherlands	2	United Kingdom*	28
Northern Ireland	1	United States	7
Norway	1	Wales	4
Portugal	2	Did not report	1

Note: Involved multiple countries within the UK.

### 3.1. Outcomes reporting

A total of 347 unique outcomes were identified in the 87 articles included in the review. Table 4 presents citations for the 87 articles. A table with each of these articles and the outcomes they presented is provided as Supplementary material. Please see this table for references for the outcomes described in the narrative sections below.

**Table 4:** Articles included in the review.

#	Original research articles
(34)	Aggar C, Thomas T, Gordon C, Bloomfield J, Baker J. Social Prescribing for Individuals Living with Mental Illness in an Australian Community Setting: A Pilot Study. Community Mental Health J. 2021;57(1):189–95.
(35)	Bertotti M, Frostick C, Findlay G, Harden A, Netuveli G, Renton A, et al. Shine 2014 Final Report: Social Prescribing: integrating GP and Community Assets for Health: UEL Research Repository [Internet]. University of East London; 2015 [cited 2023 Jul 7] p. 1–33. Available from: https://repository.uel.ac.uk/item/8962y
(36)	Bhatti S, Rayner J, Pinto AD, Mulligan K, Cole DC. Using self-determination theory to understand the social prescribing process: a qualitative study. BJGP Open. 2021 Apr;5(2):BJGPO.2020.0153.
(37)	Brettell M, Fenton C, Foster E. Linking Leeds: A Social Prescribing Service for Children and Young People. Int J Environ Res Public Health. 2022 Jan 27;19(3):1426.
(38)	Carnes D, Sohanpal R, Frostick C, Hull S, Mathur R, Netuveli G, et al. The impact of a social prescribing service on patients in primary care: a mixed methods evaluation. BMC Health Serv Res. 2017 Dec 19;17(1):835.
(39)	Cheshire A, Richards R, Cartwright T. ‘Joining a group was inspiring’: a qualitative study of service users’ experiences of yoga on social prescription. BMC Complementary Medicine and Therapies. 2022 Mar 14;22(1):67.
(40)	Dayson C, Bashir N. The social and economic impact of the Rotherham social prescribing pilot: Main evaluation report [Internet]. Sheffield, United Kingdom: Sheffield Hallam University; 2014 p. 1–63. Available from: https://shura.shu.ac.uk/18961/1/Dayson-SocialAndEconomicImpact-Rotherham%28VoR%29.pdf
(41)	Dayson C, Bennett E. Evaluation of Doncaster Social Prescribing Service: Understanding outcomes and impact [Internet]. Sheffield, United Kingdom: Sheffield Hallam University; 2016 [cited 2023 Jul 6] p. 1–34. Available from: https://www.shu.ac.uk/centre-regional-economic-social-research/publications/evaluation-of-doncaster-social-prescribing-service-understanding-outcomes-and-impact
(42)	Dayson C, Painter J, Bennett E. Social prescribing for patients of secondary mental health services: emotional, psychological and social well-being outcomes. Journal of Public Mental Health. 2020 Jan 1;19(4):271–9.
(43)	Efstathopoulou L, Bungay H. Mental health and resilience: Arts on Prescription for children and young people in a school setting. Public Health. 2021 Sep;198:196–9.
(44)	Elston J, Gradinger F, Asthana S, Lilley-Woolnough C, Wroe S, Harman H, et al. Does a social prescribing ‘holistic’ link-worker for older people with complex, multimorbidity improve well-being and frailty and reduce health and social care use and costs? A 12-month before-and-after evaluation. Prim Health Care Res Dev. 2019 Sep 24;20:e135.
(45)	Farenden C, Mitch C, Feast S, Verdenicci S. Community Navigation in Brighton & Hove Evaluation of a social prescribing pilot [Internet]. Brighton & Hove, United Kingdom: Impetus; 2015 p. 1–68. Available from: https://ihub.scot/media/1656/cn-full-evaluation-nov-2015.pdf
(46)	Foster A, Thompson J, Holding E, Ariss S, Mukuria C, Jacques R, et al. Impact of social prescribing to address loneliness: A mixed methods evaluation of a national social prescribing programme. Health Soc Care Community. 2021 Sep;29(5):1439–49.
(47)	Giebel C, Morley N, Komuravelli A. A socially prescribed community service for people living with dementia and family carers and its long-term effects on well-being. Health Soc Care Community. 2021 Nov;29(6):1852–7.
(48)	Golden TL, Maier Lokuta A, Mohanty A, Tiedemann A, Ng TWC, Mendu M, et al. Social prescription in the US: A pilot evaluation of Mass Cultural Council’s “CultureRx.” Frontiers in Public Health [Internet]. 2023 [cited 2023 Jul 6];10. Available from: https://www.frontiersin.org/articles/10.3389/fpubh.2022.1016136
(49)	Grant C, Goodenough T, Harvey I, Hine C. A randomised controlled trial and economic evaluation of a referrals facilitator between primary care and the voluntary sector. BMJ. 2000 Feb 12;320(7232):419–23.
(50)	Grayer J, Cape J, Orpwood L, Leibowitz J, Buszewicz M. Facilitating access to voluntary and community services for patients with psychosocial problems: a before-after evaluation. BMC Family Practice. 2008 May 7;9(1):27.
(51)	Hanlon P, Gray CM, Chng NR, Mercer SW. Does Self-Determination Theory help explain the impact of social prescribing? A qualitative analysis of patients’ experiences of the Glasgow “Deep-End” Community Links Worker Intervention. Chronic Illn. 2021 Sep;17(3):173–88.
(52)	Hassan SM, Giebel C, Morasae EK, Rotheram C, Mathieson V, Ward D, et al. Social prescribing for people with mental health needs living in disadvantaged communities: the Life Rooms model. BMC Health Serv Res. 2020 Jan 6;20(1):19.
(53)	Hemingway A, Jack E. Reducing social isolation and promoting well being in older people. Quality in Ageing and Older Adults. 2013;14(1):25–35.
(54)	Holt NJ. Tracking momentary experience in the evaluation of arts-on-prescription services: using mood changes during art workshops to predict global wellbeing change. Perspect Public Health. 2020 Sep 1;140(5):270–6.
(55)	Howarth M, Griffiths A, da Silva A, Green R. Social prescribing: a “natural” community-based solution. Br J Community Nurs. 2020 Jun 2;25(6):294–8.
(56)	Jones C, Hartfiel N, Brocklehurst P, Lynch M, Edwards RT. Social Return on Investment Analysis of the Health Precinct Community Hub for Chronic Conditions. Int J Environ Res Public Health. 2020 Jul 21;17(14):5249.
(57)	Kellezi B, Wakefield JRH, Stevenson C, McNamara N, Mair E, Bowe M, et al. The social cure of social prescribing: a mixed-methods study on the benefits of social connectedness on quality and effectiveness of care provision. BMJ Open. 2019 Nov 1;9(11):e033137.
(58)	Kimberlee R. Gloucestershire clinical commissioning group’s social prescribing service: Evaluation report [Internet]. Bristol, United Kingdom: University of the West of England; 2016 [cited 2023 Jul 6] p. 1–54. Available from: https://uwe-repository.worktribe.com/output/905835
#	Original research articles
(59)	Kimberlee RH, Ward R, Jones M, Powell J. Measuring the economic impact of Wellspring Healthy Living Centre’s Social Prescribing Wellbeing Programme for low level mental health issues encountered by GP services [Internet]. Bristol, United Kingdom: University of the West of England; 2014 p. 1–111. Available from: https://networks.sustainablehealthcare.org.uk/sites/default/files/resources/2014_kimberlee_0.pdf
(60)	Loftus AM, McCauley F, McCarron MO. Impact of social prescribing on general practice workload and polypharmacy. Public Health. 2017 Jul;148:96–101.
(61)	Longwill A. Independent Evaluation of the Hackney WellFamily Service [Internet]. London, United Kingdom: Family Action; 2014 p. 1–8. Available from: https://www.family-action.org.uk/content/uploads/2014/07/Wellfamily-Evaluation-Summary.pdf
(62)	Lynch M, Jones CR. Social prescribing for frequent attenders in primary care: An economic analysis. Frontiers in Public Health. 2022;10:902199.
(63)	Makanjuola A, Lynch M, Hartfiel N, Cuthbert A, Wheeler HT, Edwards RT. A Social Return on Investment Evaluation of the Pilot Social Prescribing EmotionMind Dynamic Coaching Programme to Improve Mental Wellbeing and Self-Confidence. Int J Environ Res Public Health. 2022 Aug 26;19(17):10658.
(64)	Maughan DL, Patel A, Parveen T, Braithwaite I, Cook J, Lillywhite R, et al. Primary-care-based social prescribing for mental health: an analysis of financial and environmental sustainability. Prim Health Care Res Dev. 2016 Mar;17(2):114–21.
(65)	Mercer SW, Fitzpatrick B, Grant L, Chng NR, McConnachie A, Bakhshi A, et al. Effectiveness of Community-Links Practitioners in Areas of High Socioeconomic Deprivation. Ann Fam Med. 2019 Nov;17(6):518–25.
(66)	Moffatt S, Steer M, Lawson S, Penn L, O’Brien N. Link Worker social prescribing to improve health and well-being for people with long-term conditions: qualitative study of service user perceptions. BMJ Open. 2017 Jul 1;7:e015203.
(67)	Moore EJ, Thew M. Exploring the perspectives of ‘young adults’ (18–24) who have been in formal care and their experiences of attending a socially prescribed community allotment gardening group. British Journal of Occupational Therapy. 2023 Jan 1;86(1):26–32.
(68)	Morton L, Ferguson M, Baty F. Improving wellbeing and self-efficacy by social prescription. Public Health. 2015 Mar;129(3):286–9.
(69)	Payne K, Walton E, Burton C. Steps to benefit from social prescription: a qualitative interview study. Br J Gen Pract. 2020 Jan;70(690):e36–44.
(70)	Pescheny JV, Gunn LH, Randhawa G, Pappas Y. The impact of the Luton social prescribing programme on energy expenditure: a quantitative before-and-after study. BMJ Open. 2019 Jun 1;9(6):e026862.
(71)	Polley M, Fixen A, Seers H. Evaluation of a Social Prescribing Pilot in Shropshire – implementation and impact findings. European Journal of Integrative Medicine. 2021;48:101949.
(72)	Prior F, Coffey M, Robins A, Cook P. Long-Term Health Outcomes Associated With an Exercise Referral Scheme: An Observational Longitudinal Follow-Up Study. J Phys Act Health. 2019 Apr 1;16(4):288–93.
(73)	Redmond M, Sumner RC, Crone DM, Hughes S. “Light in dark places”: exploring qualitative data from a longitudinal study using creative arts as a form of social prescribing. Arts Health. 2019 Oct;11(3):232–45.
(74)	Simpson S, Smith S, Furlong M, Ireland J, Giebel C. Supporting access to activities to enhance well-being and reduce social isolation in people living with motor neurone disease. Health Soc Care Community. 2020 Nov;28(6):2282–9.
(75)	South J, Higgins TJ, Woodall J, White SM. Can social prescribing provide the missing link? PHC. 2008;9(4):310–8.
(76)	Stickley T, Eades M. Arts on prescription: a qualitative outcomes study. Public Health. 2013 Aug;127(8):727–34.
(77)	Stickley T, Hui A. Social prescribing through arts on prescription in a U.K. city: participants’ perspectives (part 1). Public Health. 2012 Jul;126(7):574–9.
(78)	Sumner RC, Crone DM, Baker C, Hughes S, Loughren EA, James DVB. Factors associated with attendance, engagement and wellbeing change in an arts on prescription intervention. J Public Health (Oxf). 2020 Feb 28;42(1):e88–95.
(79)	Sumner RC, Crone DM, Hughes S, James DVB. Arts on prescription: observed changes in anxiety, depression, and well-being across referral cycles. Public Health. 2021 Mar;192:49–55.
(80)	Thomson LJ, Lockyer B, Camic PM, Chatterjee HJ. Effects of a museum-based social prescription intervention on quantitative measures of psychological wellbeing in older adults. Perspect Public Health. 2018 Jan;138(1):28–38.
(81)	Thomson LJ, Morse N, Elsden E, Chatterjee HJ. Art, nature and mental health: assessing the biopsychosocial effects of a “creative green prescription” museum programme involving horticulture, artmaking and collections. Perspect Public Health. 2020 Sep;140(5):277–85.
(82)	Vogelpoel N, Jarrold K. Social prescription and the role of participatory arts programmes for older people with sensory impairments. Journal of Integrated Care. 2014 Jan 1;22(2):39–50.
(83)	Wakefield JRH, Kellezi B, Stevenson C, McNamara N, Bowe M, Wilson I, et al. Social Prescribing as ‘Social Cure’: A longitudinal study of the health benefits of social connectedness within a Social Prescribing pathway. J Health Psychol. 2022 Feb 1;27(2):386–96.
(84)	White J, Kinsella K, South J. An evaluation of social prescribing health trainers in south and west Bradford. Leeds, United Kingdom: Leeds Metropolitan University; 2010 Dec p. 1–39.
(85)	Wigfield A, Kispeter E, Alden S, Turner R, Clarke T. Age UK’s fit for the future project [Internet]. Leeds, United Kingdom: University of Leeds; 2015 p. 1–112. Available from: https://www.ageuk.org.uk/globalassets/age-uk/documents/reports-and-publications/evaluation-reports/fit-for-the-future-project---final-evaluation-report-july-2015.pdf
#	Original research articles
(86)	Wildman J, Wildman JM. Evaluation of a Community Health Worker Social Prescribing Program Among UK Patients With Type 2 Diabetes. JAMA Netw Open. 2021 Sep 1;4(9):e2126236.
(87)	Wildman J, Wildman JM. Impact of a link worker social prescribing intervention on non-elective admitted patient care costs: A quasi-experimental study. Soc Sci Med. 2023 Jan;317:115598.
(88)	Wildman JM, Moffatt S, Steer M, Laing K, Penn L, O’Brien N. Service-users’ perspectives of link worker social prescribing: a qualitative follow-up study. BMC Public Health. 2019 Jan 22;19(1):98.
(89)	Wood CJ, Polley M, Barton JL, Wicks CL. Therapeutic Community Gardening as a Green Social Prescription for Mental Ill-Health: Impact, Barriers, and Facilitators from the Perspective of Multiple Stakeholders. Int J Environ Res Public Health. 2022 Oct 20;19(20):13612.
(90)	Wood E, Ohlsen S, Weich S, Fenton SJ. Understanding Social Prescribing for People With Comorbid Mental and Physical Health Conditions. A Realist Evaluation. International Journal of Qualitative Methods. 2020;19:56.
(91)	Woodall J, Trigwell J, Bunyan AM, Raine G, Eaton V, Davis J, et al. Understanding the effectiveness and mechanisms of a social prescribing service: a mixed method analysis. BMC Health Serv Res. 2018 Aug 6;18(1):604.
(92)	Zhu E, Ahluwalia JS, Laws MB. An Evaluation of Connect for Health: A Social Referral Program in RI. R I Med J (2013). 2020 Jun 1;103(5):65–9.
(93)	Healthy Dialogues [Internet]. 2023 [cited 2023 Jul 6]. Merton Social Prescribing Programme Evaluation – Healthy Dialogues. Available from: https://healthydialogues.co.uk/case_study/social-prescribing_v2/
Protocol articles	
(94)	Dingle GA, Sharman LS, Hayes S, Chua D, Baker JR, Haslam C, et al. A controlled evaluation of the effect of social prescribing programs on loneliness for adults in Queensland, Australia (protocol). BMC Public Health. 2022 Jul 19;22(1):1384.
(95)	Halder MM, Wakefield JR, Bowe M, Kellezi B, Mair E, McNamara N, et al. Evaluation and exploration of a social prescribing initiative: Study protocol. J Health Psychol. 2021 Mar;26(3):345–56.
(96)	Hoffmeister LV, Nunes MF, Figueiredo CEM, Coelho A, Oliveira MFF, Massano P, et al. Evaluation of the Impact and Implementation of Social Prescribing in Primary Healthcare Units in Lisbon: A Mixed-Methods Study Protocol. Int J Integr Care. 2021 Jun 21;21(2):26.
(97)	Kiely B, Clyne B, Boland F, O’Donnell P, Connolly D, O’Shea E, et al. Link workers providing social prescribing and health and social care coordination for people with multimorbidity in socially deprived areas (the LinkMM trial): protocol for a pragmatic randomised controlled trial. BMJ Open. 2021 Feb 1;11(2):e041809.
(98)	Moffatt S, Wildman J, Pollard TM, Penn L, O’Brien N, Pearce MS, et al. Evaluating the impact of a community-based social prescribing intervention on people with type 2 diabetes in North East England: mixed-methods study protocol. BMJ Open. 2019 Jan 15;9(1):e026826.
(99)	Wallace S, Wallace C, Elliott M, Davies M, Pontin D. Enhancing higher education student well-being through social prescribing: a realist evaluation protocol. BMJ Open. 2022 Mar 1;12(3):e052860.
Review articles	
(25)	Araki K, Takahashi Y, Okada H, Nakayama T. Social prescribing from the patient’s perspective: A literature review. Journal of General and Family Medicine. 2022;23(5):299–309.
(16)	Bickerdike L, Booth A, Wilson PM, Farley K, Wright K. Social prescribing: less rhetoric and more reality. A systematic review of the evidence. BMJ Open. 2017 Apr 7;7(4):e013384.
(27)	Bild E, Pachana NA. Social prescribing: A narrative review of how community engagement can improve wellbeing in later life. Journal of Community & Applied Social Psychology. 2022;32(6):1148–215.
(33)	Chatterjee HJ, Camic PM, Lockyer B, Thomson LJM. Non-clinical community interventions: a systematised review of social prescribing schemes. Arts & Health. 2018 May 4;10(2):97–123.
(21)	Cooper M, Avery L, Scott J, Ashley K, Jordan C, Errington L, et al. Effectiveness and active ingredients of social prescribing interventions targeting mental health: a systematic review. BMJ Open. 2022 Jul 1;12(7):e060214.
(18)	Costa A, Sousa CJ, Seabra PRC, Virgolino A, Santos O, Lopes J, et al. Effectiveness of Social Prescribing Programs in the Primary Health-Care Context: A Systematic Literature Review. Sustainability. 2021 Jan;13(5):2731.
(26)	Hamilton-West K, Milne A, Hotham S. New horizons in supporting older people’s health and wellbeing: is social prescribing a way forward? Age Ageing. 2020 Apr 27;49(3):319–26.
(28)	Helitzer E, Clements-Cortes A, Moss H. Group singing on social prescription: A scoping review: Singing on social prescription. Music and Medicine. 2022 Oct 30;14(4):226–37.
(31)	Husk K, Blockley K, Lovell R, Bethel A, Lang I, Byng R, et al. What approaches to social prescribing work, for whom, and in what circumstances? A realist review. Health Soc Care Community. 2020 Mar;28(2):309–24.
(22)	Kiely B, Croke A, O’Shea M, Boland F, O’Shea E, Connolly D, et al. Effect of social prescribing link workers on health outcomes and costs for adults in primary care and community settings: a systematic review. BMJ Open. 2022 Oct 17;12(10):e062951.
#	Review articles
(23)	Kilgarriff-Foster A, O’Cathain A. Exploring the components and impact of social prescribing. Journal of Public Mental Health. 2015 Jan 1;14(3):127–34.
(30)	Liebmann M, Pitman A, Hsueh YC, Bertotti M, Pearce E. Do people perceive benefits in the use of social prescribing to address loneliness and/or social isolation? A qualitative meta-synthesis of the literature. BMC Health Services Research. 2022 Oct 19;22(1):1264.
(32)	Little M, Rosa E, Heasley C, Asif A, Dodd W, Richter A. Promoting Healthy Food Access and Nutrition in Primary Care: A Systematic Scoping Review of Food Prescription Programs. Am J Health Promot. 2022 Mar;36(3):518–36.
(15)	Napierala H, Krüger K, Kuschick D, Heintze C, Herrmann WJ, Holzinger F. Social Prescribing: Systematic Review of the Effectiveness of Psychosocial Community Referral Interventions in Primary Care. Int J Integr Care. 22(3):11.
(14)	Percival A, Newton C, Mulligan K, Petrella RJ, Ashe MC. Systematic review of social prescribing and older adults: where to from here? Fam Med Community Health. 2022 Oct;10(Suppl 1):e001829.
(17)	Pescheny JV, Randhawa G, Pappas Y. The impact of social prescribing services on service users: a systematic review of the evidence. Eur J Public Health. 2020 Aug 1;30(4):664–73.
(5)	Polley MJ, Whiteside J, Elnaschie S, Fixsen A. What does successful social prescribing look like? Mapping meaningful outcomes [Internet]. London, United Kingdom: University of Westminster; 2020 Feb [cited 2023 Jul 6] p. 1–61. Available from: https://42b7de07-529d-4774-b3e1-225090d531bd.filesusr.com/ugd/14f499_5f193389d80c4503a4c800e026189713.pdf
(19)	Reinhardt GY, Vidovic D, Hammerton C. Understanding loneliness: a systematic review of the impact of social prescribing initiatives on loneliness. Perspect Public Health. 2021 Jul;141(4):204–13.
(24)	Rempel ES, Wilson EN, Durrant H, Barnett J. Preparing the prescription: a review of the aim and measurement of social referral programmes. BMJ Open. 2017 Oct 1;7(10):e017734.
(29)	Thomas T, Aggar C, Baker J, Massey D, Thomas M, D’Appio D, et al. Social prescribing of nature therapy for adults with mental illness living in the community: A scoping review of peer-reviewed international evidence. Front Psychol. 2022;13:1041675.
(20)	Vidovic D, Reinhardt GY, Hammerton C. Can Social Prescribing Foster Individual and Community Well-Being? A Systematic Review of the Evidence. Int J Environ Res Public Health. 2021 May 15;18(10):5276.

#### 3.1.1. Most frequently reported unique outcomes

Eight unique outcomes were studied or reported in 10 or more articles and were identified as the most commonly studied unique outcomes (See Table 5). Also notably, weight and BMI were reported in 8 articles.

**Table 5:** Most studied unique outcomes.

Most Frequently Reported Unique Outcomes (≥10)	# of Articles
Overall wellbeing	19
Confidence	16
Social isolation	16
General practitioner (GP) Visits	14
Anxiety	11
Physical activity	11
Depression	11
Loneliness	10

#### 3.1.2. Most frequently reported outcome categories

**Figure 2:**
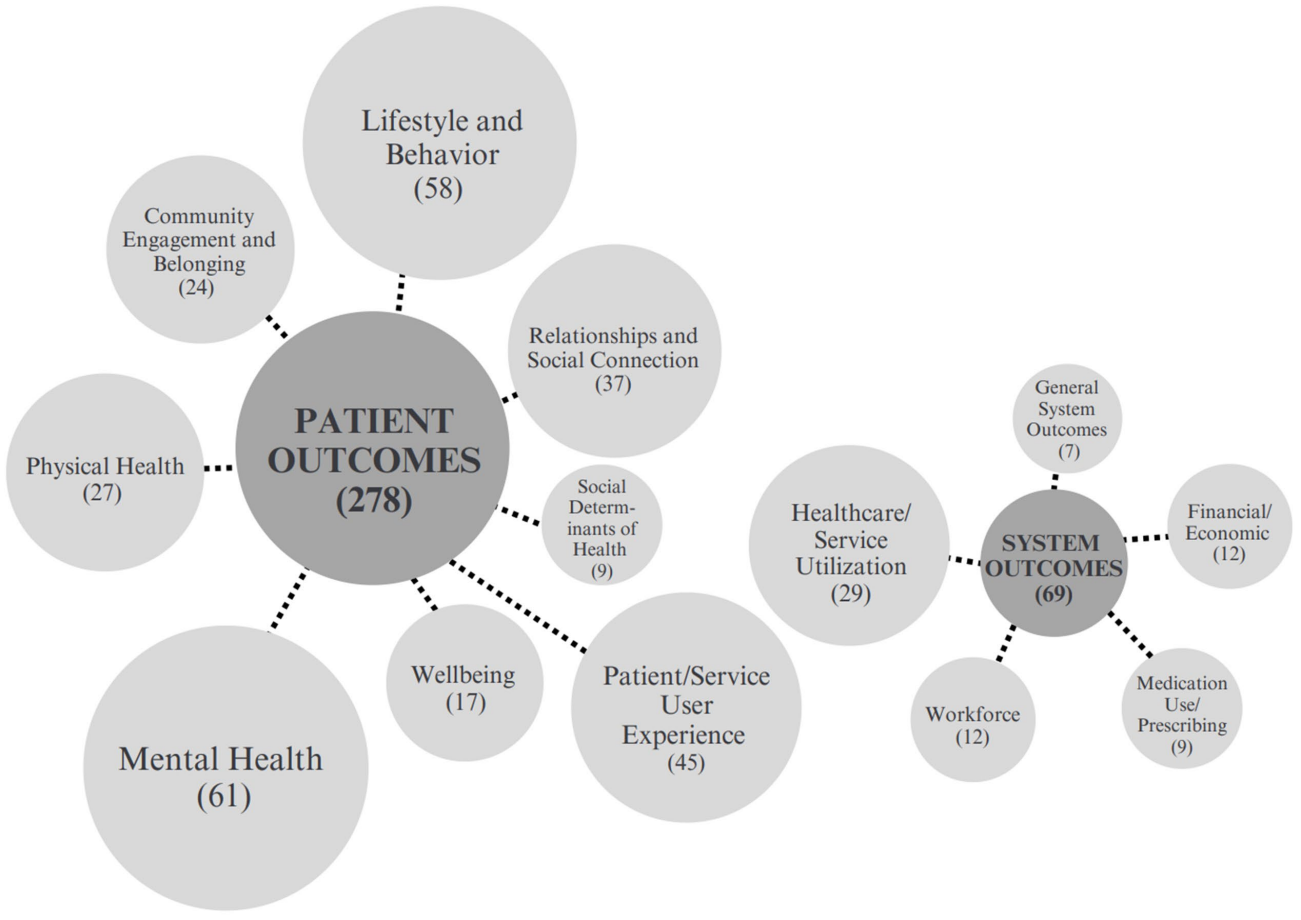
Outcome categories.

Given the variation in terminologies used for outcomes across the articles, same and similar outcomes were grouped into categories to better represent outcome interests across the studies. These categories of outcomes present a more comprehensive view of the outcomes studied in the 87 articles (See Figure 2). The following sections present quantitative and qualitative descriptions of the outcomes found in each category, including how often the more common outcomes were reported, and examples of notable characteristics of some of the included studies. Figures are included for outcomes categories with numerous sub-categories.

#### 3.1.3. Patient outcomes

The review identified a total of 278 unique patient outcomes. The highest prevalence of outcomes fell under the category of mental health, with nearly as many in the lifestyle and behavior category (Table 6).

**Table 6:** Patient level outcomes.

Patient-level outcomes	Number of unique outcomes in the category
Mental health	61
Lifestyle and behavior	58
Patient/service user experience	45
Relationships and social connection	37
Physical health	27
Community engagement and belonging	24
Wellbeing	17
Social determinants of health	9
Total	278

##### 3.1.3.1. Mental health

**Figure 3:**
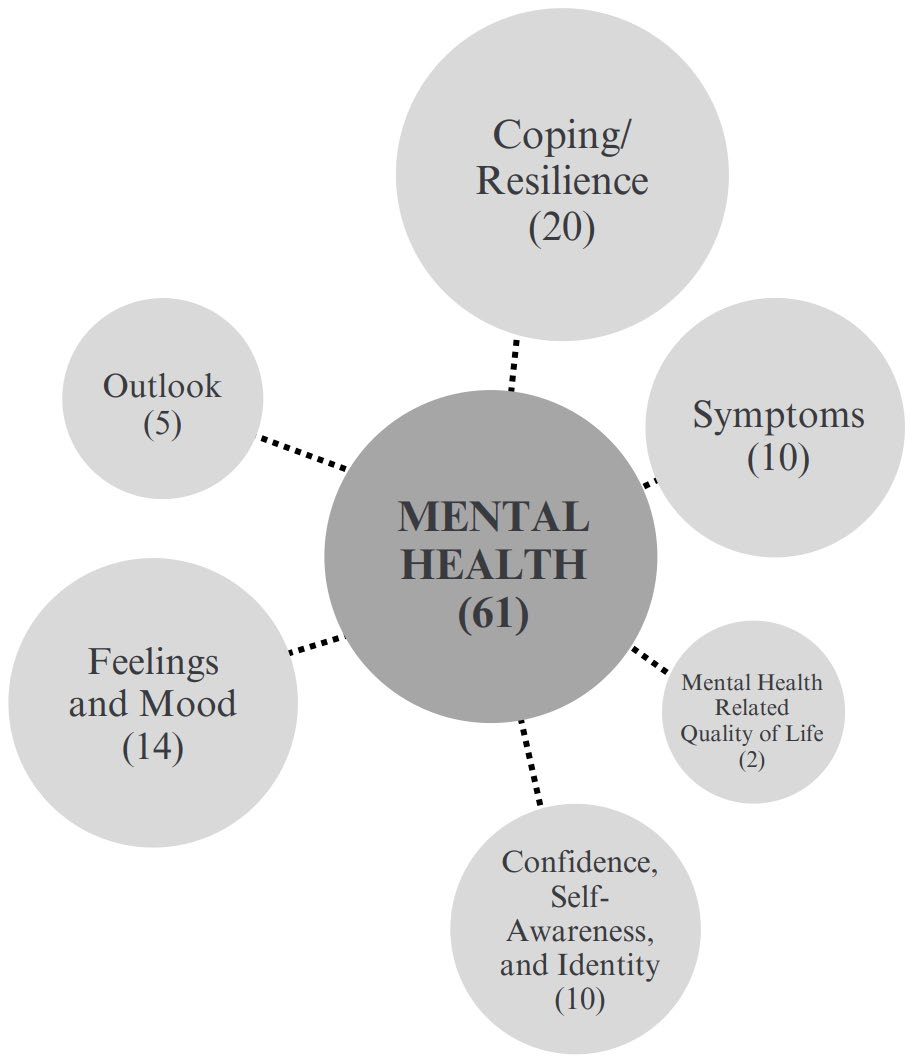
Mental health outcomes sub-categories.

The mental health category encompasses 61 unique outcomes and was the largest category of outcomes. Figure 3 presents a set of 6 sub-categories that encompass these 61 mental health outcomes. Mental health outcomes were studied in 49 of the 66 original research articles and protocols included in this review. It was also reported on in all but one of the 21 review articles.

In this category, the most frequently studied unique outcomes were mental well-being in 19 articles, confidence in 16 articles, anxiety in 11 articles, loneliness in 10 articles, depression in 11 articles, and overall mental health in 8 articles. Other commonly studied unique mental health outcomes included mental health related quality of life, dimensions of mood, identity, and sense of self. Figure 3 presents a set of 6 sub-categories that encompass these 61 mental health outcomes.

In a study utilizing the UCL Museum Wellbeing Measure at pre- and post-intervention, Thomson et al. (81) studied outcomes related to mental health among patients who had engaged in a combined program of horticulture and arts-based activities. Similarly, Dayson and Bennett (41) used a mixed-methods approach including interviews and diaries to investigate mental health outcomes related to a social prescribing service over a one-year period, and Foster et al. (46) assessed the impact of a social prescribing intervention developed and delivered by the British Red Cross to decrease loneliness using the UCLA 3-item Loneliness scale and interviews to assess changes in loneliness between demographic groups.

##### 3.1.3.2. Lifestyle and behavior

**Figure 4:**
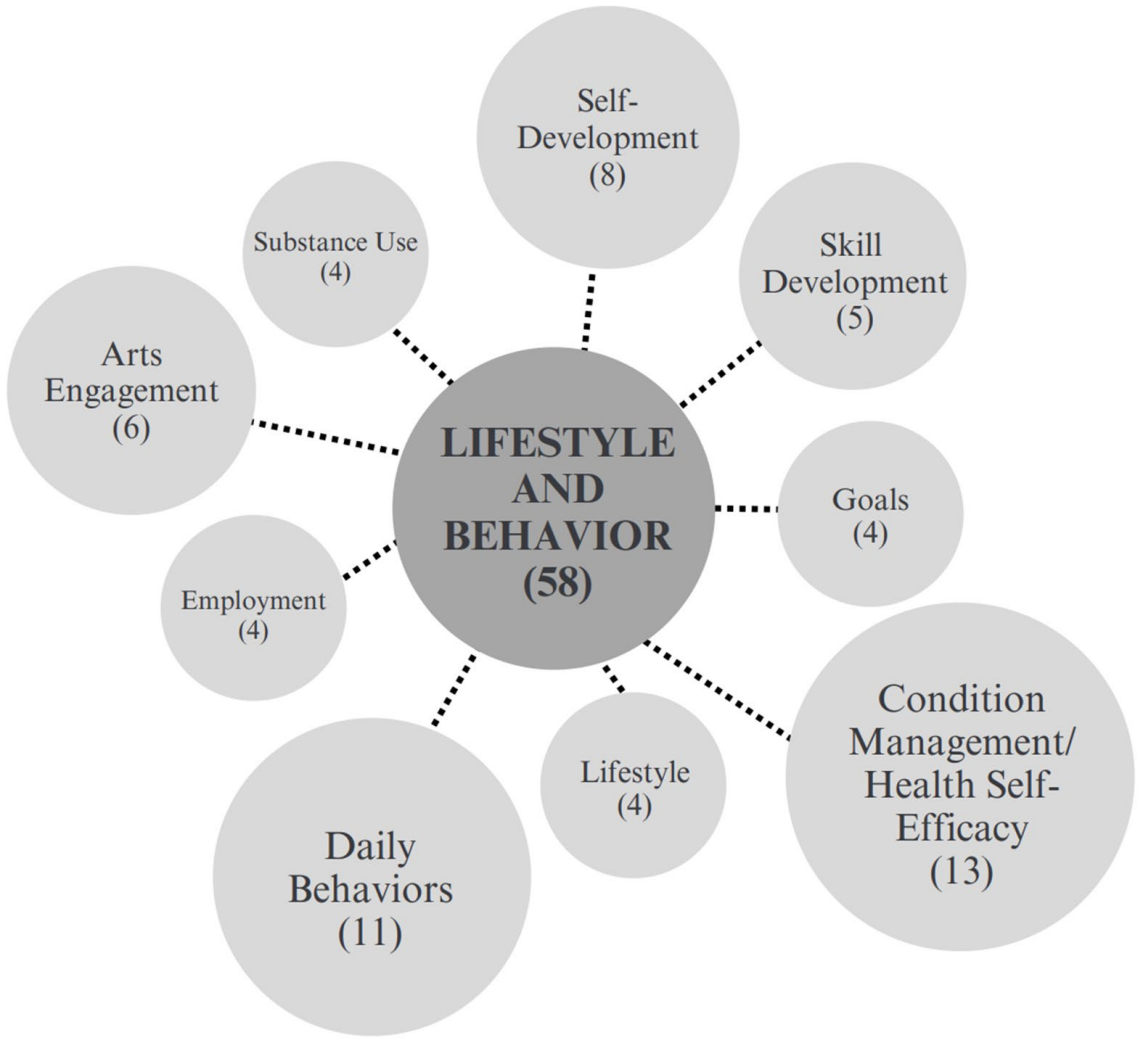
Lifestyle and behavior sub-categories.

The second largest category of outcomes was lifestyle and behavior. Among the 56 outcomes presented across 33 papers, the most frequently occurring were self-management in 7 studies, patient activation in 5 studies, smoking status in 4 studies, alcohol consumption in 4 studies, independence in 4 studies, and skill development in 4 studies. This category included 9 sub-categories, as shown in Figure 4.

Self-management, the most commonly studied outcome in this category, is defined as the “tasks that individuals must undertake to live well with one or more chronic conditions” (100). Among the 7 studies that measured self-management, 3 included interviews (36, 45, 89), 1 utilized focus groups (52) and 2 performed both interviews and focus groups (36, 89). Questionnaires were also utilized in 2 studies (35, 95), and one used the Patient Activation Measure (97). It is notable that among the populations studied in relation to self-management, 2 studies reported working with community-dwelling adults with multimorbidities (44, 97).

Patient activation, which refers to the “skills and confidence a person has in managing their own health and health care” Lynch and Jones (62), was noted as relevant to social prescribing due its link to health behaviors, clinical outcomes, and cost for delivering care. Of the 5 studies that measured patient activation, all utilized pre and post questionnaires for data collection, 4 included interviews (71, 95, 96) or focus groups (96) and 4 used the Patient Activation Measure 13 (PAM13), a 13-statement questionnaire exploring patients’ beliefs and confidence around the management of their individual conditions (44, 71, 95, 96). Skill development was measured in 4 studies, all of which conducted semi-structured interviews followed by thematic analysis (35, 42, 52, 67).

##### 3.1.3.3. Patient/service user experience

**Figure 5:**
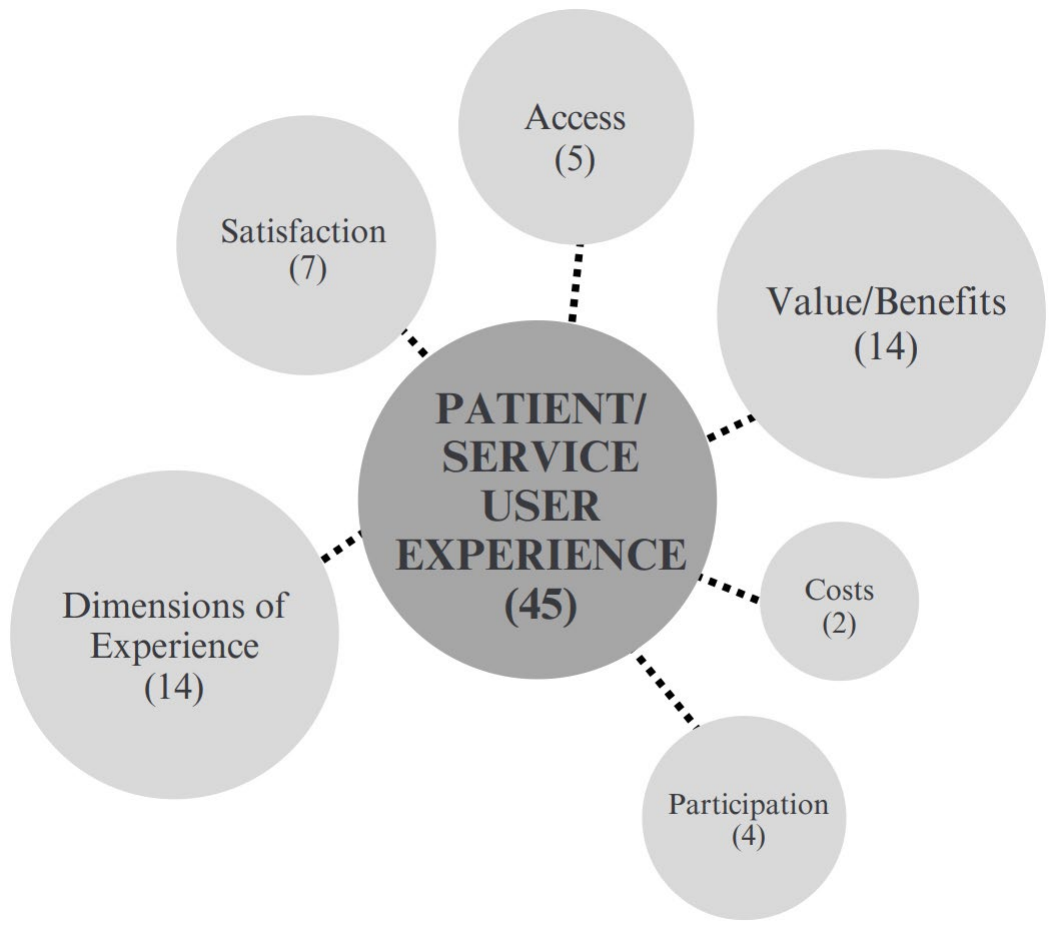
Patient and service user experience.

The patient/service user experience category included 45 unique outcomes presented across 24 papers and organized into 6 sub-categories (See Figure 5). Enjoyment, as a social prescribing user experience, was measured in 14 papers, and patient satisfaction was reported in 8 papers. Ten different outcomes related to relationships with the service provider were reported in six papers and two papers reported on health and social cost to patients. Four papers reported on program quality and three reported on attendance in SP activities. Additionally, there were four outcomes concerned with accessibility, including access to social, emotional, and practical support, access for people with mental health issues, and access related to mobility issues such as transport, equipment provision, and using mobility aids in a community home environment.

Simpson et al. (74) analyzed findings of a pilot service based in England using a thematic analysis after interviewing people living with motor neuron disease and link workers post-intervention. Hanlon et al. (51) also employed a thematic analysis of semi-structured interviews with 12 patients in Scotland referred to Community Links Practitioners using a Self-Determination Theory. Distinctly, Hoffmeister et al. (96) presented an evaluation protocol for the first SP program in Portugal. They embarked on a mixed-methods approach that entailed a longitudinal, prospective study with data collected via questionnaires by patients at four time-points. In addition, secondary data was collected on medical records and both interviews and focus groups were conducted with key stakeholders.

##### 3.1.3.4. Relationships and social connections

**Figure 6:**
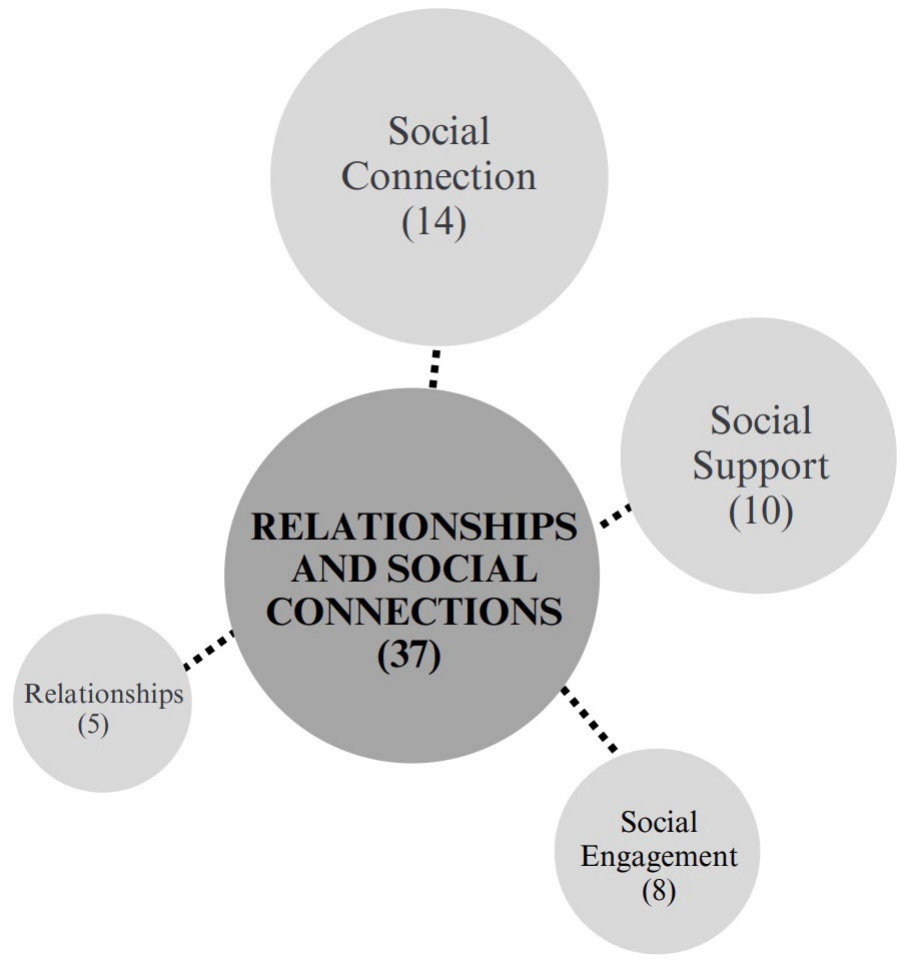
Relationships and social connections.

The relationships and social connections category was organized into 4 subcategories, as shown in Figure 6. Social connection and social support were the most significant of those sub-categories. Across 36 different outcomes studied and reported on in 33 papers, the most common unique outcome studied was social isolation, which was measured in 16 studies. The next most common outcomes were social connectedness which was reported in 7 articles, and social connection which was reported in 5. Other commonly studied outcomes included social networks, which was reported on in 4 articles, social relationships in 4 articles, and group membership, also in 4 articles. Alongside these outcomes are the inclusion of reported social support in 4 articles and friendship which was reported across 5 studies. Reconnection and social engagement were also reported in two studies each.

Notably, when Moore et al. (67) explored the thoughts of young adults (18–24) in a social prescribing gardening group, they found that all participants described a sense of social connection, not only within the group itself, but also in the local community. Another qualitative study exploring social isolation among older adults (53) reported that participants felt particularly benefited by the friendship of their peers, whether new or maintained, and being able to have a shared world view with someone.

##### 3.1.3.5. Physical health

In the physical health category, 28 different physical outcomes were reported across 23 articles. The most common unique outcome studied was physical activity, which was measured in 11 studies. Studies that assessed physical activity among program participants tracked dimensions of activity, such as frequency (56) and intensity (85). Weight and BMI were measured in 8 studies and blood pressure was measured in 3 studies. Moffatt et al. (98) presented a quasi-experimental mixed-method study protocol for evaluating changes in glycated hemoglobin, weight, cholesterol, and smoking status using Secondary Uses Service and Quality Outcomes Framework data, and ethnographic methods, including observation, interviews and focus groups, to observe how patients engage with social prescribing. Other physical health outcomes studied included sleep, energy, pain, and mobility.

##### 3.1.3.6. Community engagement and belonging

The community engagement and belonging category was comprised of 24 unique outcomes, including belonging, social belonging, sense of community, community identification, and community connection. Among the 24 outcomes reported across 19 articles, belonging was reported 3 times.

For example, Moore and Thew (67) reported that feeling a sense of belonging, not just within the social prescribing activity group itself but also with their local community, was one of the most important motivators for engaging in community allotment programs. Additionally, Wakefield et al. (83) documented that a sense of belonging allowed individuals to feel that social support is available from others, thereby helping them feel less lonely. Hassan et al. (52) documented how lack of community-based social care opportunities result in patients looking for social support from public health and how SP brought patients a sense of social belonging. Stickley and Eades (76) reported that the structure of the community-based program enhanced the patient’s experience by providing social support. Golden et al. (48) reported on an evaluation of a state-level arts prescribing program that included 12 pilot sites. The evaluation found that enhanced community connection was a benefit for participating patients as well as for healthcare providers as they perceived it as a way to increase their care capacity.

##### 3.1.3.7. Wellbeing

The wellbeing category is composed of 17 unique outcomes that address various aspects of wellbeing and which were reported across 44 articles. The most commonly measured outcome was mental well-being, which was reported in 19 articles. Some articles reported on other specific aspects of well-being, such as physical well-being, social well-being, emotional well-being, personal well-being, and psychological well-being.

Additional outcomes in the wellbeing category included quality of life, which was reported in 9 articles and general health, which was reported in 6 studies. Among those measuring general health, 4 conducted interviews while 6 utilized surveys and questionnaires with tools such as the Dartmouth COOP/WONCA functional health assessment chart (49) and the World Health Organization QoL tool (WHOQoL) (34). Giebel et al. (47) explored the effects of social prescribing on individuals with dementia and family caregivers in England by measuring participants’ wellbeing at baseline and at three and six-month follow-up periods.

##### 3.1.3.8. Social determinants of health

The social determinants of health (101) category included 9 different outcomes, reported in 9 articles, including housing in 2 studies, employment and support with work in 3 studies, and access to resources in 1 study. A significant area of inquiry in this category was related to welfare services, including welfare needs, awareness of welfare benefits, and access to wider welfare benefits. One study also measured access to resources and management of social determinants of health by employing interviews with patients and providers (92). In a study that tested prospective findings against published findings from a systematic search, Payne et al. (69) assessed participants’ perception of their personal assets and their future.

#### 3.1.4. System-level outcomes

The review identified a total of 69 unique system outcomes. The highest prevalence of outcomes (29) fell under the category of healthcare and service utilization. Financial and economic outcomes were also commonly measured, as were outcomes related to workforce. Other commonly studied outcomes included financial and/or economic, workforce, medication use/prescribing, and general system outcomes (Table 7).

**Table 7:** System level outcomes.

System-level outcomes	*N*
Healthcare and service utilization	29
Financial/economic	12
Workforce	12
Medication use/prescribing	9
General system outcomes	7
Total	69

##### 3.1.4.1. Healthcare/service utilization

**Figure 7:**
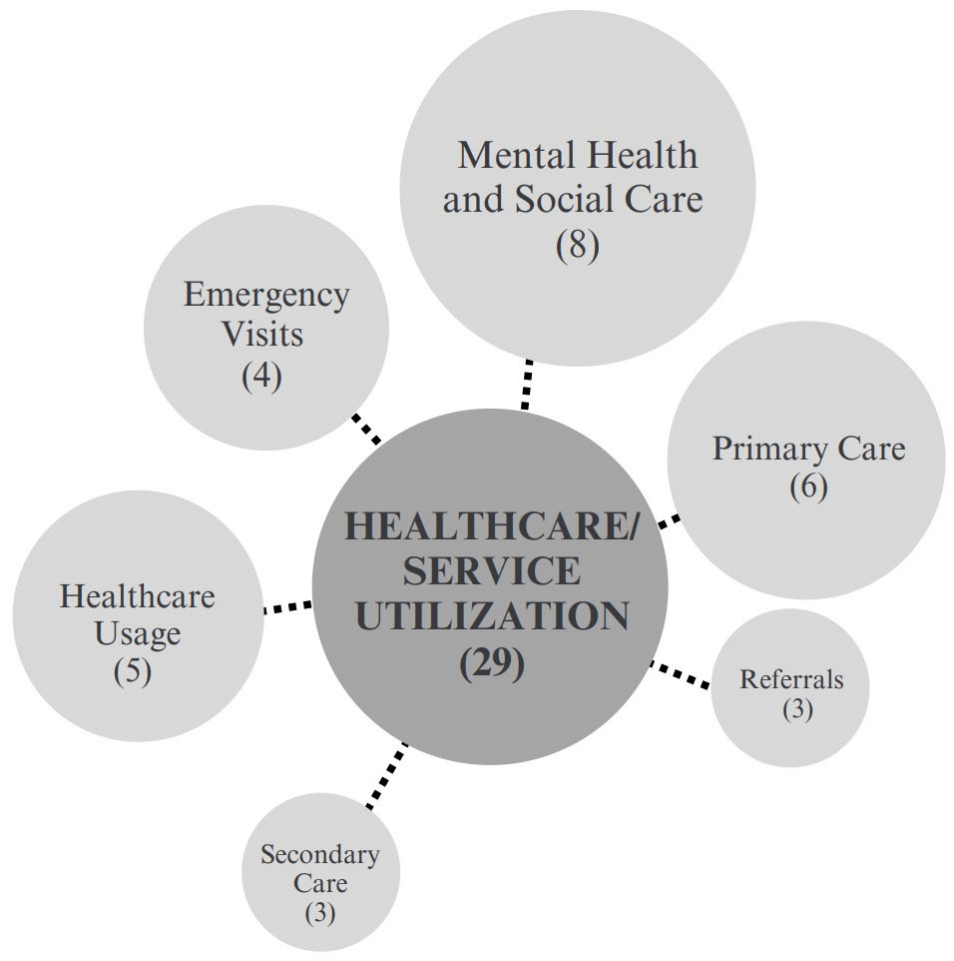
Healthcare/service utilization sub-categories.

Among 29 unique outcomes identified across 26 papers and organized into 6 sub-categories, the most studied in the healthcare/service utilization category was mental health and social care utilization. This outcome was reported in 8 articles. Other unique outcomes included clinical referrals, length of stay and time spent with care providers. Visits to general or primary care practitioners and emergency service visits were also reported across 10 articles (Figure 7).

Other studies outcomes included number of hospital admissions in 2 articles, inpatient admissions and stays in 2 articles, outpatient encounters in 3 articles, and nurse visits in 2 articles. South et al. (75), considered how the social prescribing intervention extended primary care by offering a public health intervention and building health alliances. Referrals were also assessed in 3 articles. Notably, methods for measuring healthcare utilization were variability across studies, including patient self-reports and analyzes of administrator-driven patient records.

##### 3.1.4.2. General system outcomes

The general system outcomes category captures 7 outcomes reported across 5 papers that relate more generally or holistically to the healthcare system. For example, within a comprehensive program evaluation, Farenden et al. (45) assessed health equality, integration of services, and institutional partnerships formed using patient interviews and surveys with both volunteers and general practitioners (GPs). Other articles reported general outcomes such as the expansion of care options, group based psychological resources (94) and general practitioner recognition of need for change in health services.

##### 3.1.4.3. Medication Use and prescribing

One of the smaller categories of outcomes was medication use and prescribing. This category encompasses 9 unique outcomes reported across 10 papers, and including medication use, medication consumption, prescription for all drugs, psychotropic medication use, and anti-depressant compliance. Also included in this category were studies of the number of prescriptions dispensed, number of patients with no new repeat medications, number of medications, and number and type of regularly prescribed medications. For example, Kiely et al. (97) published a protocol for a pragmatic randomized controlled trial designed to assess an array of outcomes, including the number and type of regularly prescribed medications.

##### 3.1.4.4. Financial/economic outcomes

The financial and economic outcomes category included 12 unique outcomes, which were reported in 14 articles. One of the more common outcomes in this category was Social Return on Investment (SROI), which assigns monetary value to social outcomes, and was reported in 5 articles. This category also included cost savings per participant reported in 1 study, total care costs reported in 2 studies, cost per patient in 2 studies, health cost savings in 2 studies, and the leveraging of funding from additional sources, financial savings, carbon savings, and psychotropic medication costs, which were each reported in 1 study.

For example, Maughan et al. (64) reported on how social prescribing services can reduce financial burdens and lower environmental costs of health care. Wildman and Wildman (87) studied the effect of social prescribing interventions on patients in areas of high socioeconomic deprivation. From a holistic perspective, Lynch and Jones (62) studied the economic benefits arising from changes in healthcare resources after implementing social prescribing interventions.

##### 3.1.4.5. Workforce

The workforce category includes 13 unique outcomes reported across 9 papers, and related to workforce experiences, perceptions, and outcomes. Workforce members include caregivers, volunteers, and staff. Outcomes measured in this category include staff turnover, volunteering, volunteer well-being, caregiver well-being, and link workers’ experiences. Each of these outcomes was reported in 1 study.

For example, Simpson et al. (74) studied training needs and how link workers were employed through the service of social prescribing through a co-design, while Longwill (61) measured staff turnover and knowledge of staff. Other single studies assessed prescriber well-being, prescriber work experience, and provider workload.

## 4. Discussion

This mapping review identified, categorized, and described a broad array of outcomes that have been studied in relation to social prescribing programs in the 13 countries cited in the World Health Organization’s Social Prescribing Toolkit (2). It identified 347 unique outcomes, including 278 patient-level outcomes (e.g., mental health, lifestyle and behavior, community engagement and belonging) and 69 systems-level outcomes (e.g., healthcare/service utilization, medication use/prescription). It identified the most frequently studied unique outcomes, as well as the most frequently studied categories of patient- and system-level outcomes. This work builds on and advances previous work undertaken in the UK that has identified and collated program outcomes studied in that nation, where social prescribing has been operating informally for over three decades but has been formally part of the National Health Service (NHS) delivery since 2019. While many of the outcomes and outcome categories identified in this mapping review align with that of the previous work undertaken by Polley et al. (4, 5), this review identified a wider range of outcomes that represent a wider geographic area of programming as well as a more recent period of time in which more programming has been implemented and more research and evaluation undertaken.

### 4.1. Mental health

Mental health was the most frequently studied outcome area across the articles included in this review. Strong interest in mental health outcomes aligns with the international mental health crisis which has been exacerbated by the COVID-19 pandemic (102), and with the aims of social prescribing programs to address and promote mental health across the lifespan (103, 104). The range of 61 unique mental health outcomes found in this review reflect the nascent stage of research on social prescribing, but also offer nuanced insight into how the broad range of social prescribing programs that are being implemented affect a wide range of dimensions of mental health. This heterogeneity may be positive in regard to the study of outcomes across diverse populations, whose lived experiences and priorities vary greatly, making a variety of measures necessary to addressing mental health more equitably and with the nuanced insight it both requires and deserves. However, this heterogeneity-and that represented across other outcome categories in this review - poses significant challenges to evidence synthesis, and particularly to opportunities for meta-analysis of specific outcomes that can help advance evidence-based practice and policy. To date, very few systematic reviews or meta-analyzes exist to guide practice, research, policy, or investment in social prescribing, and the evidence base is consistently referred to as lacking in quality (16). These circumstances limit advancement of promising practices, as well as investment and policy that could make the benefits of social prescribing more available to individuals and health systems.

### 4.2. Relevance to emerging research priorities: loneliness and social isolation

This review identified outcomes that, given their relationship to critical health and social issues, warrant both broader and deeper study. Two categories–community engagement/belonging and relationships/social connection - are together concerned with outcomes related to loneliness and social isolation or connection. This area of study aligns with the growing understanding of the impacts that loneliness and social isolation have on health outcomes (105), and highlights the potential for social prescribing programs to play a role in addressing these critical issues. Further research that explores this potential is highly warranted.

### 4.3. Non-communicable diseases

Another emerging topic of research was non-communicable or chronic diseases (NCDs), which are responsible for 74% of all deaths globally and 86% of premature deaths in middle- and low-income countries (106). This review highlights numerous outcomes related to opportunities for better outcomes and management related to NCDs that could be afforded by social prescribing. Outcomes in the physical health and lifestyle and behavior categories, namely outcomes such as patient activation, self-management, social connection, support, and coping may be frequently measured due to their link to health behaviors, clinical outcomes, and cost for delivering care. Additionally, the prevalence of outcomes related to physical activity, weight, and BMI suggests potential for social prescribing programs to help address epidemics of obesity in many nations as well. NCD deaths are often linked to health behaviors and health management skills, and have also been studied in relation to environmental risk factors such as disasters (107), as well as international aid and country wealth (108). Identifying growth outcome areas such as these may embolden public and systems understanding that urgent public health issues such as chronic disease, mental health, collective trauma, racism, and social exclusion and isolation are challenges that social prescribing, and especially programs that include arts and culture, can help address (109). Additionally, social prescribing research should consider individuals living with disabilities and working toward disability justice–what writer, poet Naomi Ortiz defines as “a cross-disability (sensory, intellectual, mental health/psychiatric, neurodiversity, physical/mobility, learning, etc.) framework that values access, self-determination and an expectation of difference” (110, 111).

### 4.4. Health equity

Some attention is being given to how social prescribing can potentially help address and advance health equity, as well as to its potential to exacerbate health disparities (112, 113). However, in this review, very few studies addressed or examined health equity. One article presented health equality as an outcome (45), and control of health, which connects with the understanding of health equity being defined as “the attainment of the highest level of health for all people” (114) was explored in 1 study (39). Though access to services were addressed in several articles, an explicit focus on measuring equity was not clear. There has been recent debate as to the potential of social prescribing to reduce health inequalities. The aim is part of some countries’ core principles in developing social prescribing schemes as part of developments in personalized care, patient empowerment, reducing healthcare pressures, and addressing key social determinants of health (115, 116). However, social prescribing only addresses some of the causes of health inequalities, which is compounded by the fact that the same social factors that affect people’s health can also impact their capacity to engage with social prescribing, meaning that even well-intentioned social prescribing programs could inadvertently disproportionately benefit the healthier, widening the gap in health disparities (117). Nonetheless, there is promise from case studies of well-targeted social prescribing programs, and a greater focus on assessment of outcomes related to equity and health equity is critical.

An important aspect of examining the relationship of social prescribing to health equity is the collection of sociodemographic information. The Institute for Healthcare Improvement recommends not only collecting sociodemographic characteristics of individuals, but also calls for better calculation of stratified measures of disparities, which include opportunities to observe within-group differences in addition to between-group differences, such as Asian subpopulations (e.g., Chinese, Indian) and black subpopulations (e.g., US-born Black vs. Haitian vs. Nigerian)” (118). One of the studies in this review (45) noted that equalities monitoring data for patients is not consistently collected by primary care services and made a recommendation inclusion of specialists in equalities to be engaged in social prescribing research. Furthering this idea, future social prescribing research should scaffold the foundations of data collection with measures specific to health equity and intercultural justice. Resources such as the Health Equity Measurement Framework (119) and Health Equity Measurement Framework for Medicaid Accountability (118, 120) can inform these decisions. In the UK, indices of multiple deprivation (IMD) are frequently used to collect granular demographic information in social prescribing research. However, this approach, and even the concept of deprivation, is not common in some other nations such as the United States. As public health in some areas shifts from a focus on social determinants of health to social drivers of health, social need screenings should address these social drivers of health, namely food insecurity, housing instability, transportation problems, utility needs, and interpersonal safety. This would require cultural shifts in both programming and research practices to evolve from over-studying the experiences of predominantly White populations that fail to include or reflect the lived experience of People of the Global Majority using better typologies of health equity measures (121).

### 4.5. Implications for future evidence synthesis

This review identified several challenges to searching the social prescribing outcomes literature as well as in the reporting of outcomes. One challenge was that, at the time that this search was conducted, social prescribing was not consistently defined or described in the literature. Subsequently however, Muhl et al. (1) created a highly useful set of internationally accepted conceptual and working definitions for social prescribing. This work has great promise for advancing reporting on social prescribing as well as for advancing the precision of future evidence synthesis. To date, many publications have failed to report on the involvement of clinicians, link workers, and referral processes, highlighting the need for development of reporting guidelines for social prescribing outcomes research. Additionally, this review identified search terms that can be problematic in relation to social prescribing. For example, the term social referral is used in relation to social prescribing and is also a marketing term used to describe the phenomenon by which people refer a product to someone else. Terms used in relation to primary care and general practitioner services (e.g., GP surgeries, GP attendance, primary care visits) also vary widely across countries. As healthcare service utilization is a common and important area of study, search strategies must be inclusive of a variety of terms. Lastly, many of the outcomes identified in this review, such as user experience and social relationships, are not easily measured through quantitative means. This highlights the need for mixed methods research designs that can capture both quantitative and qualitative dimensions of social prescribing and its outcomes.

### 4.6. Strengths and limitations

There were several strengths and limitations in this review. A primary strength was that the review was able to use and build on search strategies developed by research teams in the UK, with permission and input from the authors (4, 5). Another significant strength of the study was the wide scope of inquiry that the mapping review methodology allowed. The review was able to include protocols, reviews, and studies utilizing any research methods, as well as program evaluations and reports. The inclusion of reviews helped to ensure that the search strategy was effective and allowed consideration of how other researchers had considered, quantified, and categorized outcomes. This review was not duplicative of previous reviews, which were generally focused on different or smaller geographic areas. Additionally, the review sought to categorize unique outcomes from the literature which presents patterns and trends in an easily comprehensible manner. This review took care to document outcomes in the bespoke ways in which they were studied and to which they were referred (specific language) in the respective publications. This approach allowed for precision and inclusion of a wide array of concepts and concerns related to social prescribing outcomes across the 13 nations. It provided the opportunity for development of more granular categories of outcomes and for a wider articulation of the impacts of social prescribing interventions than have previously been published. Finally, the categorization process undertaken in this review was important, as many of the unique outcomes identified were very similar in nature, and often referred to the same concepts in different terms. As such, the categorization presented offers meaningful suggestions for outcomes that could be prioritized in future studies to advance the potential for evidence synthesis, which is critical to advancing evidence-based practice, policy, and investment in this promising area of practice.

One limitation of the review was the inconsistency in definitions used for social prescribing which posed a significant challenge when screening articles, and to a lesser extent, through the extraction process. While the topic of social prescribing is understandably nascent, general consensus includes some “prescribing” aspect in a traditionally clinical environment, meaning that even before a link worker or equivalent professional is involved, a healthcare worker of some kind is the impetus for a patient accessing a community-based activity. Several terms used in the articles were confounding, including “community referral” and “social referral.” Due to the lack of consistency in terminology related to social prescribing and its component parts, the study team was left to discern what qualified as social prescribing. As a result, some relevant articles may have been excluded. Further, this review may not have captured the breadth of current work on social prescribing as a large portion of research into social prescribing goes unpublished or is documented in the restricted format of reports by a private company or health system. Finally, this mapping review did not undertake a critical appraisal process. As such, the relative quality of each study may not reflect a high level of rigor. This aligns with calls from the field for more rigorous studies and more systematic processes (16).

## 5. Conclusion

From a synthesis of research conducted in 13 countries, this mapping review has shown that social prescribing has relevance to over 300 health and health system outcomes, and that outcomes related to mental health, lifestyle, and behavior are most frequently studied. The review highlights the need for more complex study designs that can take account of multiple outcome measures across diverse populations. It contributes to the advancement of evidence synthesis for social prescribing globally by quantifying and offering insight into the outcomes that have been studied to date and by laying a foundation for the development of key common outcomes and a Core Outcomes Set, both of which will be critical to increasing precision and quality in social prescribing research. While breadth in outcomes research is essential to measurement and relevance across diverse health needs in different populations and parts of the world, consistency in measurement of key common outcomes is also essential to building the potential for meta-analysis and, in turn, evidence-based practice and policy.

## Data availability statement

The original contributions presented in the study are included in the article/Supplementary material, further inquiries can be directed to the corresponding author.

## Author contributions

JS: Conceptualization, Data curation, Formal analysis, Investigation, Methodology, Project administration, Writing – original draft, Writing – review & editing, Resources, Supervision, Validation, Visualization. NM: Writing – review & editing, Conceptualization, Data curation, Formal analysis, Investigation, Methodology, Project administration, Validation. CB: Conceptualization, Data curation, Formal analysis, Investigation, Methodology, Project administration, Supervision, Validation, Writing – original draft, Writing – review & editing. JM-D: Conceptualization, Investigation, Methodology, Writing – review & editing, Writing – original draft. SA: Investigation, Writing – original draft, Writing – review & editing, Data curation, Formal analysis. SM: Data curation, Formal analysis, Investigation, Writing – original draft, Methodology. OO: Data curation, Formal analysis, Investigation, Writing – original draft. GH: Data curation, Formal analysis, Investigation, Writing – original draft, Project administration, Supervision. GM: Data curation, Formal analysis, Investigation, Writing – original draft. GD: Data curation, Formal analysis, Investigation, Writing – original draft, Methodology, Validation. AR: Data curation, Formal analysis, Investigation, Methodology, Writing – original draft, Conceptualization, Project administration, Writing – review & editing. SB: Validation, Writing – original draft, Writing – review & editing. AC: Validation, Writing – original draft, Writing – review & editing. VP: Supervision, Validation, Writing – original draft, Writing – review & editing. DF: Writing – review & editing, Validation.

## Supplementary material

SUPPLEMENTARY TABLE 1 Citations by patient outcomes.

SUPPLEMENTARY TABLE 2 Search strategy.

SUPPLEMENTARY FIGURE 1 Systems-level outcomes reported by country.

SUPPLEMENTARY FIGURE 2 Patient-level outcomes reported from each country.
