# Supplementary material for: Social prescribing outcomes: a mapping review of the evidence from 13 countries to identify key common outcomes
Source: Front Med (Lausanne). 2023 Nov 7;10:1266429. doi: 10.3389/fmed.2023.1266429 (PMC10660286; doi:10.3389/fmed.2023.1266429)
Supplement: SUPPLEMENTARY TABLE 1 — Citations by patient outcomes. [file Table_1.DOCX]

| **Outcome** | **Citation # (see reference list in manuscript)** |
| --- | --- |
| **PATIENT OUTCOMES** |  |
| **Well-being** |  |
| Overall well-being | (35,38,40,44–47,51,53,59,61,65,66,71,74,93–95,97) |
| Personal well-being | (37,99) |
| Sense of well-being | (45) |
| Mental well-being | (37,43,44,46,54,55,58,59,63,67,68,72,78,79,82,85,91,96,99) |
| Physical well-being | (67,85) |
| Social well-being | (42,85) |
| Emotional well-being | (42) |
| Psychological well-being | (34,42,80,81) |
| Life satisfaction | (37, 85) |
| Health related quality of life | (35,65,72,97) |
| Positive and active engagement in life | (35,38) |
| Quality of life | (34,41,42,45,61,83,95,96,98) |
| Overall/general health | (34,35,38,49,53,71,91,94) |
| Economic well-being | (59) |
| Biopsychosocial well-being | (34) |
| Health status | (34,56) |
| Environmental quality of life | (34) |
| **Mental Health** |  |
| Anxiety | (35,37,38,41,49,59,61,65,68,79,91) |
| Confidence | (41,42,46,51,52,55,56,66,68,69,71,74,77,89–91) |
| Happiness | (37,42) |
| Hope for the future | (35,89) |
| Loneliness | (40,41,46,52,57,71,83,85,94,95) |
| Mental health | (36,39,50,84,88–90,97) |
| Coping | (41,42,66,88) |
| Depression | (35,38,41,49,59,61,65,68,79,90,91) |
| Hope | (69,91) |
| Mood | (39,54,67,71) |
| Psychological distress | (50,61) |
| Self-worth | (89) |
| Optimism | (37,91) |
| Self-esteem | (46,48,76,81,88) |
| Empowerment | (48,90) |
| Resilience | (43,66,69,89,99) |
| Self-efficacy | (48,63,67,68,95) |
| Self-confidence | (39,57,66,67,76,82,88) |
| Motivation | (76,77) |
| Sense of control | (45,51,66,95) |
| Autonomy | (42,51,77) |
| Feeling positive | (40,85) |
| Emotional loneliness | (71) |
| Ability to avoid crisis | (41) |
| Identity | (61) |
| Mental health related quality of life | (90) |
| Positive affect | (42) |
| Self-acceptance | (42) |
| Psychological quality of life | (34) |
| Thinking clearly | (37) |
| Ability to deal with problems | (37) |
| Feeling relaxed | (37) |
| Ability to navigate through complex situations | (41) |
| Stress management | (39) |
| Purpose | (36,42,68,69,89,91) |
| Stigma | (74) |
| Mental health symptoms | (61) |
| Emotional problems | (61) |
| Supported discussions in therapy | (48) |
| Psychological impact on daily living | (51) |
| Feelings of non-judgement | (73) |
| Competence | (51) |
| Self-awareness | (52) |
| Tense arousal | (54) |
| Capability | (97) |
| Outlook | (69) |
| Mood - inspired | (81) |
| Mood of - happy | (81) |
| Mood - excited | (81) |
| Mood - enthusiastic | (81) |
| Mood - alert | (81) |
| Mood - active | (81) |
| Stressors | (89) |
| Distraction from mental illness | (89) |
| Patient-reported burden of treatment | (97) |
| Feelings | (49) |
| Sense of being oneself | (73) |
| Solace | (89) |
| Putting life into perspective | (89) |
| Self-perception | (69) |
| Positive outlook | (76) |
| **Physical Health** |  |
| Fraility | (44) |
| Cholesterol | (71,98) |
| Diet | (85,88) |
| Weight/ body mass index | (39,66,71,72,84,85,96,98) |
| Energy | (39,48) |
| Blood pressure | (71) |
| Mobility | (41,55) |
| Blood glucose | (66,86) |
| Pain | (48,49) |
| Physical activity | (40,56,59,65,66,70–72,85,88,96) |
| Sleep | (39) |
| Systolic blood pressure | (72,98) |
| Type of chronic diseases | (96) |
| Physical health | (90) |
| Feeling Looser, more limber, less stiff | (48) |
| Physical quality of life | (34) |
| Flexibility | (38) |
| Balance | (38) |
| Posture | (38) |
| Strength | (38) |
| General fitness | (38) |
| Daily consumption of fruit and vegetable intake per day | (85) |
| Waist circumference | (85) |
| Physical problems |  |
| Increase in energy expenditure from walking | (70) |
| Increase in energy expenditure from moderate activity | (70) |
| Increase in energy expenditure from vigorous activity | (70) |
| **Community Engagement and Belonging** |  |
| Social belonging | (52,77) |
| Sense of community | (36,81) |
| Community connection | (48) |
| Community belonging | (57,95) |
| Belonging | (67,82,83) |
| Community engagement | (40,88) |
| Community links | (45,61) |
| Community cohesion | (45) |
| Group cohesion | (82) |
| Perception of safety with environment | (67) |
| Feeling of safety within environment | (67) |
| Collective efficacy | (95) |
| Community uplift | (48) |
| Increase awareness and access to community arts | (48) |
| Feeling left out | (37) |
| Economic participation | (34) |
| Sense of social integration | (76) |
| New group identification | (94) |
| Social inclusion | (52) |
| Group identities | (94) |
| Leisure center membership | (56) |
| Community care | (44) |
| Activity group identification | (95) |
| Community identification | (57,95) |
| **Relationships and Social Cohesion** |  |
| Social connection | (36,48,56,57,67) |
| Social connectedness | (39,41,52,59,61,89,91) |
| Social engagement | (69,90) |
| Social networks | (35,37,42,91) |
| Positive relationships | (42,51) |
| Friendship | (53,67,82,89,90) |
| Social isolation | (37,40–42,45,52,55,59,66,81,82,84,85,90,91,95) |
| Social relationships | (34,37,53,89) |
| Reconnection | (35,48) |
| Group membership | (57,83,94,95) |
| Social support | (83,89,95) |
| Interactions with local people | (35) |
| Relating | (69) |
| Being able to talk things through with someone and feeling reassured | (71) |
| Feeling supported | (71) |
| Confidant support | (49) |
| Affective support | (49) |
| Shift in social perceptions (trust, empathy, inclusion) | (76) |
| Lack of companionship | (37) |
| Social impact on daily living | (51) |
| Romantic relationships | (53) |
| Social recovery | (61) |
| Peer-support | (90) |
| Relatedness | (51) |
| Social activity | (45,49) |
| Beneficence | (51) |
| Former and new group compatibility | (94) |
| Professional support | (69) |
| Interpersonal connections | (91) |
| Feeling close to other people | (37) |
| Social opportunities | (77) |
| Quality of family relationships | (76) |
| Social contact | (76) |
| Being with others | (73) |
| Social functioning | (90) |
| Social benefits | (77) |
| **Lifestyle and Behavior** |  |
| Smoking status | (71,72,96,98) |
| Smoking rates | (65,85) |
| Alcohol consumption | (65,72,85,96) |
| Lifestyle change | (39,84) |
| Development of interests | (42,52) |
| Independence | (40,52,69,91) |
| Self-management | (36,37,40,45,52,89,97) |
| Control of care | (39,51,85) |
| Goal attainment | (44,61,95) |
| Goal setting | (66,95) |
| Achievement | (67,68) |
| Condition management | (40,88) |
| Lifestyle score | (40) |
| Work, volunteering, and other activities | (40) |
| Structure to life | (35) |
| Work and social adjustments | (65) |
| New interests | (42) |
| Health self-efficacy | (34) |
| Ability to make positive choices | (45) |
| Change in behavior | (51) |
| Health-promoting behaviors | (69) |
| Weekly activity | (95) |
| Knowledge and ability a person has to improve their health | (71) |
| Number of chronic diseases | (96) |
| Relationship to art making | (82) |
| Art skill development | (82) |
| Productivity | (67) |
| Return to employment | (58) |
| Daily activities | (49) |
| Awareness of personal health needs | (34) |
| Interest in arts/culture | (48) |
| Skill development | (35,42,52,67) |
| Self-development | (42,52) |
| Feel more supported to manage their long-term conditions | (85) |
| Activities of daily living | (97) |
| Smoking cessation | (88) |
| Allowing achievement | (73) |
| Health-related behaviors | (66,91) |
| Being on my own | (73) |
| Identity claims as an artist | (76) |
| Artistic interest | (76) |
| Requiring support with managing money | (37) |
| Ability to self-manage long term condition | (88) |
| Greater intrinsic regulation of behavior | (51) |
| Regulation of behavior | (51) |
| Practical skills | (89) |
| Assertiveness | (76) |
| Communication skills | (76) |
| Social skills | (77,89) |
| Improved long-term condition management | (66) |
| Decision making | (37) |
| Knowledge of health | (39) |
| Skills and competence in the job market | (45) |
| Interest of arts and crafts | (76) |
| Patient activation | (44,62,71,95,96) |
| Aspiration to return to work | (76) |
| **Patient/ Service User Experience** |  |
| Participation in SP program | (61,74) |
| Escape | (67,73) |
| Service satisfaction | (38,40,45) |
| Listened to | (45,57,71) |
| Health costs to participants | (44,95) |
| Valuable | (53,82) |
| Useful/ worthwhile | (35,37) |
| Healthcare experience for people with mental illness | (61) |
| Gained access to the right information to help address their issue | (45) |
| Adherence to the activities | (96) |
| Accepted and planned activities | (96) |
| Patient experiences | (96) |
| Patient concerns | (71) |
| Integrated approach | (61) |
| Opportunity for play | (73) |
| Doing something for me (take time for myself) | (73) |
| Activity group attendance | (95) |
| Barriers to participation- home adaptations | (74) |
| Self-expression | (77) |
| Artistic skills | (77) |
| Learning | (69) |
| Artistic development | (76) |
| Allowed them to voice their priorities | (51) |
| Comfortable | (90) |
| Perception of meaningful use of time | (37) |
| Class suitability | (39) |
| Relationship with intervention instructor | (39) |
| Gratitude | (48) |
| Activity group support | (95) |
| Relationship with link worker/ health coach | (57) |
| Service quality | (61) |
| Would recommend the service | (38) |
| Safe and reliable service | (61) |
| Accessible service | (61) |
| Relationship with service provider | (95) |
| User involvement | (61,68) |
| Need for social services support | (37) |
| Service is accountable and outcome oriented | (61) |
| Practical support and can-do attitudes | (61) |
| Connection with nature and outdoors | (89) |
| Opportunity for change, growth or coming to terms with a given situation | (73) |
| Social care costs to participants | (44,95) |
| Barriers to participation- mobility | (74) |
| Threshold moments | (73) |
| Ability to access SP services | (45) |
| **Social Determinants of Health** |  |
| Employment | (35,59) |
| Housing | (37,90) |
| Access to wider welfare benefits | (40) |
| Awareness of welfare benefits | (40) |
| Welfare needs | (34) |
| Support with work | (84) |
| Management of social determinants of health for the patients | (92) |
| Access to resources | (92) |
| Perception of personal assets and future | (69) |
| **SYSTEM OUTCOMES** |  |
| **Healthcare/ Service Utilization** |  |
| Accident and emergency visits | (35,40,41,44,58,93,96) |
| Inpatient admissions | (40,41,44) |
| Use of specialist services | (61) |
| Outpatient encounters | (40,44,97) |
| GP consultation | (35,38) |
| GP time | (84) |
| Healthcare Use | (64,71) |
| GP contact (General Practitioner visits- in paper) | (41,50,56,58–60,62,64,71,84,91,93,96,97) |
| Health service use | (94,95) |
| Number of hospital admissions | (96,97) |
| Primary care use | (50,57,90) |
| Nurse visits | (41,71) |
| Secondary care use | (98) |
| Length of stay | (97) |
| Secondary care referrals | (64) |
| Psychosocial consultations | (50) |
| Mental health referrals | (50) |
| Mental/ emotional distress intervention | (50) |
| Psychotherapy appointments | (41) |
| Referrals to other agencies | (49) |
| Avoidance of missed healthcare appointments | (46) |
| Mental health services | (41) |
| Health and social care use | (44) |
| Extending primary care by public health intervention | (75) |
| Appropriate health service use | (90) |
| Building health alliances | (75) |
| Access to support beyond statutory services | (46) |
| Social prescribing use | (90) |
| **General System Outcomes** |  |
| Contributes to health equality | (45) |
| Supports partnership between health, voluntary, and community sectors | (45) |
| General practitioner recognition of need for change in health services | (57) |
| Integration of services | (45) |
| Group-based psychological resource | (94) |
| Professional partnerships | (75) |
| Care options | (48) |
| **Medication Use/ Prescribing** |  |
| Medication use | (89) |
| Number of prescriptions dispensed | (62) |
| Number of patients with no new repeat medications | (60) |
| Number and type of regularly prescribed medications | (97) |
| Anti-depressant compliance | (90) |
| Number of medications | (38) |
| Prescription for all drugs | (49) |
| Psychotropic medication use | (50,61) |
| Medication consumption | (96) |
| **Finance/ Economic** |  |
| Non-elective care costs | (87) |
| Cost saving per participant | (62) |
| Total costs | (44,49) |
| Cost per patient | (35,45,64) |
| Psychotropic medication costs | (64) |
| Social return on investment (SROI) | (40,46,56,61,63) |
| Health cost savings | (45,87) |
| Mean attendance cost | (58) |
| Leveraging funding from additional sources | (41) |
| Financial savings | (64) |
| Carbon savings | (64) |
| **Workforce** |  |
| Staff turnover | (61) |
| Navigator transition from volunteer to staff | (45) |
| Volunteer well-being | (46) |
| Well-being for caregivers | (47) |
| Link worker experience | (74) |
| Training needs | (74) |
| Skilled and knowledgeable workforce | (61) |
| Management and supervision | (61) |
| Volunteering | (90) |
| Prescriber well-being | (48) |
| Prescriber work experience | (48) |
| Provider workload | (92) |
