# Supplementary material for: Social prescribing outcomes: a mapping review of the evidence from 13 countries to identify key common outcomes
Source: Front Med (Lausanne). 2023 Nov 7;10:1266429. doi: 10.3389/fmed.2023.1266429 (PMC10660286; doi:10.3389/fmed.2023.1266429)
Supplement: SUPPLEMENTARY TABLE 2 — Search strategy. [file Table_2.DOCX]

Appendix A

| Line # | Search Terms |
| --- | --- |
| 1 | (“social prescrib*”[tiab] OR “social prescription*”[tiab] OR “community referral*”[tiab] OR “social referral*”[tiab] OR “art prescription*”[tiab] OR “arts prescription*”[tiab] OR “nature prescription*”[tiab]) |
| 2 | (Australia*[all fields] OR Canad*[all fields] OR Ireland[all fields] OR Irish[all fields] OR Japan*[all fields] OR “New Zealand*”[all fields] OR Portug*[all fields] OR Singapore*[all fields] OR “United Kingdom”[all fields] OR “Great Britain”[all fields] OR Scotland[all fields] OR Scottish[all fields] OR Wales[all fields] OR Welsh[all fields] OR England[all fields] OR English[all fields] OR China[all fields] OR Chinese[all fields] OR “United States”[all fields] OR US[all fields] OR USA[all fields] OR "Australia"[Mesh] OR "Canada"[Mesh] OR "Ireland"[Mesh] OR "United Kingdom"[Mesh] OR "Japan"[Mesh] OR "New Zealand"[Mesh] OR "Portugal"[Mesh] OR "Singapore"[Mesh] OR "China"[Mesh] OR "United States"[Mesh]) |
| 3 | (result*[tiab] OR outcome*[tiab] OR measur*[tiab] OR assess*[tiab] OR impact*[tiab] OR trial[tiab] OR trials[tiab] OR study[tiab] OR studies[tiab] OR evaluat*[tiab] OR intervention*[tiab] OR participant*[tiab] OR data[tiab] OR longitudinal[tiab] OR “follow-up”[tiab] OR “followed-up”[tiab] OR “follow up”[tiab] OR “followed up”[tiab] OR “pre-post”[tiab] OR “pre post”[tiab] OR baseline[tiab] OR “health care burden*”[tiab] OR “healthcare burden*”[tiab] OR wellbeing[tiab] OR “well being”[tiab] OR “well-being”[tiab] OR “mental health”[tiab] OR isolat*[tiab] OR “social inclusion”[tiab] OR “social determinant of health”[tiab] OR “social determinants of health”[tiab] OR SDOH[tiab] OR “quality of life”[tiab] OR QOL[tiab] OR HRQOL[tiab] OR WEMWBS[tiab] OR EQ5D[tiab] OR “EQ-VAS”[tiab] OR MYCaW[tiab] OR PAM[tiab] OR “WHO-5”[tiab] OR “PHQ-9”[tiab] OR survey*[tiab] OR questionnaire*[tiab] OR "Treatment Outcome"[Mesh] OR "Patient Outcome Assessment"[Mesh] OR "Patient Reported Outcome Measures"[Mesh] OR "Health Impact Assessment"[Mesh]) OR "Program Evaluation"[Mesh]) OR "Cost of Illness"[Mesh] OR "Mental Health"[Mesh] OR "Social Isolation"[Mesh] OR "Social Inclusion"[Mesh] OR "Social Determinants of Health"[Mesh] OR "Quality of Life"[Mesh] OR "Surveys and Questionnaires"[Mesh]) |
| 4 | #1 AND #2 AND #3 |
| 5 | #4, Filters: English, Japanese, Chinese, Spanish |
