# Supplementary figures and images for: Social prescribing outcomes: a mapping review of the evidence from 13 countries to identify key common outcomes
Source: Front Med (Lausanne). 2023 Nov 7;10:1266429. doi: 10.3389/fmed.2023.1266429 (PMC10660286; doi:10.3389/fmed.2023.1266429)

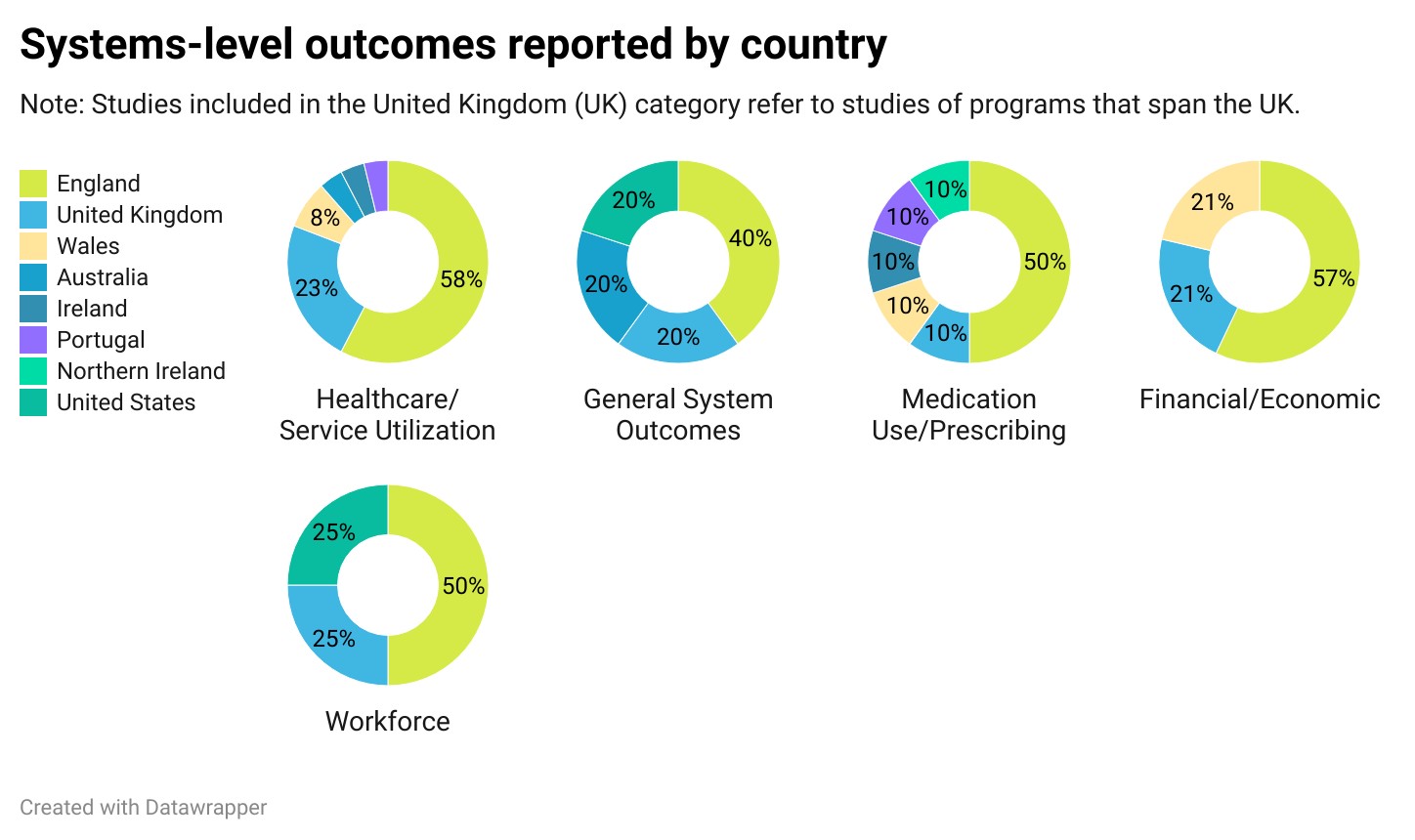

Supplement: SUPPLEMENTARY FIGURE 1 — Systems-level outcomes reported by country. [file Image_1.JPEG]

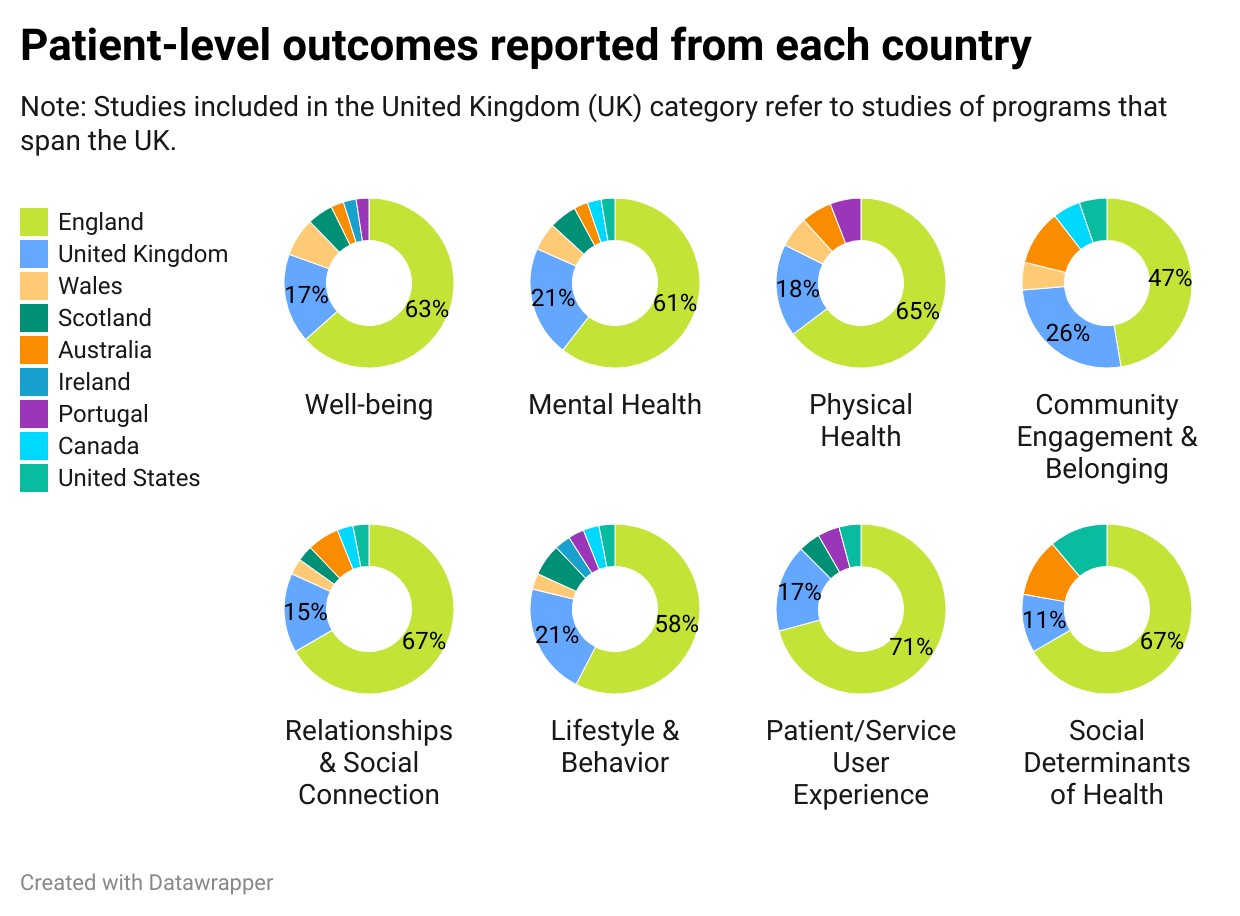

Supplement: SUPPLEMENTARY FIGURE 2 — Patient-level outcomes reported from each country. [file Image_2.JPEG]
